# 3D endothelial cell scaffolds protect liver explants and exhibit therapeutic effects on liver fibrosis

**DOI:** 10.1016/j.jhepr.2025.101617

**Published:** 2025-10-04

**Authors:** Mireia Medrano-Bosch, Alazne Moreno-Lanceta, Blanca Simón-Codina, David Saavedra-Pérez, Yiliam Fundora, Francisco J. Sánchez, Meritxell Perramón, Laura Macias-Muñoz, Manuel Morales-Ruiz, Elazer R. Edelman, Wladimiro Jiménez, Pedro Melgar-Lesmes

**Affiliations:** 1 Department of Biomedicine, School of Medicine, University of Barcelona, Barcelona, Spain; 2 Institut d'Investigacions Biomèdiques August Pi-Sunyer (IDIBAPS), Centro de Investigación Biomédica en Red de Enfermedades Hepáticas y Digestivas (CIBERehd), Barcelona, Spain; 3 Liver Transplant Unit, Institut Clínic de Malalties Digestives i Metabòliques (ICMDM), Hospital Clínic, University of Barcelona, Barcelona, Spain; 4 Biochemistry and Molecular Genetics Service, Hospital Clínic of Barcelona, Barcelona, Spain; 5 Institute for Medical Engineering and Science, Massachusetts Institute of Technology, Cambridge, MA, USA; 6 Cardiovascular Division, Brigham and Women's Hospital, Harvard Medical School, Boston, MA, USA

**Keywords:** Liver, Cell therapy, Endothelial cells, Inflammation, Fibrosis, Regeneration

## Abstract

**Background & Aims:**

Endothelial cells (ECs) display myriad protective roles that support tissue homeostasis. Embedding healthy ECs in 3D scaffolds stabilizes their phenotype to maximize reparative effects and shields immunogenicity. Here, we evaluate the protective effects of matrix-embedded ECs (MEECs) in liver explants and models of chronic liver disease.

**Methods:**

Precision cut liver slices (PCLS) from patients with cirrhosis (n = 8) and fibrotic or healthy mice (n = 6) were co-cultured with MEECs for 24 h and hepatic viability and inflammation were analyzed. The protective effects of the MEECs secretome were explored *in vitro*. MEECs were perihepatically or subcutaneously implanted for 1 week in fibrotic mice with or without hepatectomy (n = 6) to evaluate their effects on liver inflammation, regeneration, and fibrosis.

**Results:**

MEECs protected liver viability in PCLS from patients with cirrhosis (ATP/protein, 2.7 *vs.* 5.0, *p* = 0.01) and fibrotic (5.3 *vs.* 7.1, *p* = 0.01) or healthy (7.8 *vs.* 10.6, *p* = 0.01) mice, and reduced injury-induced inflammation. MEECs produced hepatocyte growth factor and fibroblast growth factor 2, which were associated with improved hepatic viability and anti-inflammatory macrophage polarization, respectively. Perihepatic implantation of MEECs in fibrotic mice with or without hepatectomy reduced inflammation and hepatic damage and exhibited pro-regenerative and antifibrotic properties (Sirius red^+^ area, 8.3 *vs.* 6.4, *p* = 0.005). These antifibrotic effects were associated with higher production of heparan sulfate and metalloproteinases 2 and 9, and mitigation of hepatic stellate cell activation. Implantation of MEECs at a distance from the liver did not reduce liver injury, inflammation, or fibrosis.

**Conclusions:**

Endothelial–hepatocyte regulation is essential in liver repair, and matrix-embedded endothelial cells (MEECs) appear to be a potential therapy for chronic liver injury and *ex situ* preservation of liver grafts.

**Impact and implications:**

Organ transplantation is the most effective therapy for advanced liver disease, yet remains limited by preservation of harvested graft viability and injury-induced inflammation post implantation. Healthy ECs display myriad protective roles that contribute to tissue homeostasis. In this study, we show how MEECs preserve cell viability and reduce inflammation in hepatic explants and display anti-inflammatory, antifibrotic and pro-regenerative properties in the liver of fibrotic mice. These dynamic and unique hepatoprotective properties of MEECs highlight their potential therapeutic utility for chronic liver injury or *ex situ* conservation of liver grafts.

## Highlights

• MEECs preserve hepatic viability and protect from injury-induced inflammation.
• MEECs ameliorate liver sinusoidal EC inflammatory phenotype.
• FGF-2 released by MEECs induces an anti-inflammatory macrophage polarization.
• MEECs reduce liver fibrosis by mitigating hepatic stellate cell activation.
• MEECs drive hepatic regeneration, in part, through the secretion of hepatocyte growth factor.

## Graphical abstract

**Figure:**
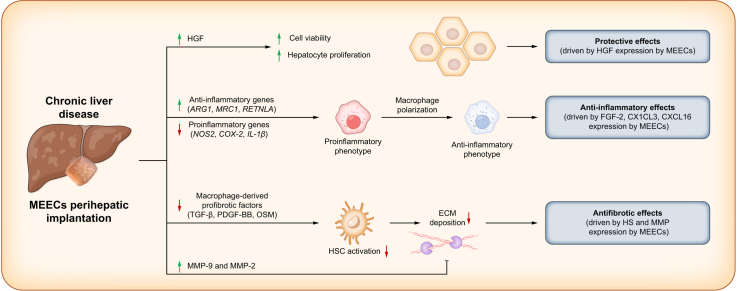

## Introduction

Liver diseases account for ∼2 million deaths annually worldwide.^1^ Liver fibrosis is the consequence of a continuous injury in the hepatic parenchyma accompanied by active extracellular matrix (ECM) remodeling with progressive and abundant deposition of collagen fibers.^2^ Baseline fibrosis limits the unique properties of hepatic self-repair and regeneration, and on-going structural and functional disorders can escalate the drive to cirrhosis and, ultimately, cancer or death. The only therapeutic alternative in patients with advanced liver disease is transplantation.^3^ However, the success of liver transplantation varies, especially in the face of graft rejection and the challenges of preserving harvested tissues and the shortage of donors. Thus, there remains an urgent need for new strategies to promote fibrosis regression and hepatic regeneration, with diverse technological advances now under investigation.[4], [5], [6]

The integrity of liver sinusoidal endothelial cells (LSECs) is crucial for controlling vascular tone, homeostasis, inflammation, and toxicant clearance.^7^ Healthy endothelial cells (ECs) maintain tissue homeostasis by regulating myofibroblastic and immune cell activity, among others.^8^^,^^9^ Acute or chronic liver injury disrupts the EC phenotype and, in doing so, negatively affects their regulatory and protective functions.^10^ Indeed, proinflammatory LSECs recruit effector immune cells through the differential expression of adhesion molecules, selectins, and chemokines.^11^ Dysfunctional LSEC phenotypes exacerbate hepatic inflammation, leading to chronic liver disease progression, and compromises the viability of liver grafts during transplantation.^12^ Retention or restoration of a healthy LSEC phenotype is important to minimize liver injury and accelerate fibrosis regression.^13^ ECs from different origins share many paracrine protective properties with specialized LSECs.^14^ ECs synthesize a significant number of basement membrane components and growth factors, including fibroblast growth factor 2 (FGF-2) and heparan sulfate (HS), which are important regulators of smooth muscle cell (SMC) and hepatic stellate cell (HSC) proliferation.^15^^,^^16^ Exogenous supplementation by direct injection or transplantation of healthy ECs can ameliorate liver cirrhosis in experimental models, but this cell therapy engenders a harmful immune rejection that limits their clinical utility.^17^

Matrix-embedded endothelial cells (MEECs) are ECs grown in a 3D collagen-based scaffold that locks EC phenotype into an energy-efficient state that retains a regulatory secretome optimized for reparative potential and shields ECs from immunogenicity *in vitro* and *in vivo* after implantation.^18^^,^^19^ MEECs can be fabricated in a reproducible fashion that enables quantifiable quality control and unit dosing, which can be preserved over time, transported intact, and implanted in specific locations. In contrast to 2D cultures of ECs, MEECs exhibit muted expression of adhesion molecules and chemokines, and a markedly decreased expression of major histocompatibility complex (MHC) class II proteins.^20^ The 3D environment mimics the physiological ultrastructure of EC–basement membrane/extracellular matrix interactions and minimizes EC stress while maximizing the secretion of regulatory factors promoting the switch of T helper (Th) 1 lymphocytes to Th2 regardless of EC origin or interspecies interactions.^14^^,^^21^ Indeed, it has been previously demonstrated that human MEECs promote a concomitant switch from proinflammatory Th1 lymphocytes and M1-like macrophages to anti-inflammatory Th2 and M2-like profiles, respectively, in healthy hepatectomized mice.^22^ MEECs express progenitor-like genes involved in angiogenesis, vasodilation, inflammation control, and tissue remodeling.^23^ The 3D assembly also shields MEECs from cytokine-induced apoptosis^24^ and the uremic milieu,^25^ conditions that are present in the hepatic microenvironment of chronic liver disease.

Therefore, we examined whether and how MEECs induce recovery of hepatic homeostasis, reduction of inflammation and fibrosis, and stimulation of regeneration in the specific complex inflammatory environment that occurs in chronic liver disease.

## Materials and methods

### Human samples

The study was approved by the Ethics Committee of the Hospital Clinic of Barcelona (reference number: HCB/2023/0532), according to the ethical guidelines of the 1975 Declaration of Helsinki. All patients included in this study provided written and signed informed consent. Human cirrhotic liver samples were obtained from liver explants of patients with end-stage cirrhosis caused by non-viral liver disease (n = 9) undergoing liver transplantation.

### Animals and *in vivo* procedures

To evaluate the protective effects of MEECs in models of chronic liver disease, male BALB/c mice (7 weeks old) were purchased from Charles River Laboratories (Charles River, Saint Aubin les Elseuf, France). All animals were maintained in a temperature-controlled room (22 °C) on a 12-h light–dark cycle. The study was performed according to the criteria of the Investigation and Ethics Committees of the Hospital Clínic and University of Barcelona and according to Animal Research: Reporting of In Vivo Experiments (ARRIVE) guidelines (reference numbers: 49/17 and 342/22). After arrival, mice were continuously fed *ad libitum* until euthanasia. To induce liver fibrosis, mice were injected intraperitoneally twice a week for 8 weeks with carbon tetrachloride (CCl_4_; ≥99.9%, Sigma-Aldrich, Merck, Darmstadt, Germany) diluted 1:8 (v/v) in corn oil (dosage: 1.25 ml CCl_4_/kg body weight). Healthy mice did not receive any treatment, to mimic the real situation of a healthy donor. Acellular matrices (AM) or MEECs were implanted between the median and right lobe of the fibrotic liver (perihepatic implantation) or on the right upper quadrant of the abdomen (subcutaneous implantation) in fibrotic mice. Partial hepatectomy (40%) was performed in fibrotic mice as previously described^22^ with brief modifications as follows: only the left lobe was fully removed in mice with liver fibrosis to avoid high numbers of deaths; AM or MEECs were implanted between the median and right lobe. The implant was held in place by the natural pressure exerted by the lobes, without the need for sutures or adhesive agents. Animals were euthanized after 1 week of treatment. Liver and blood samples were collected and frozen for further analysis. Liver restoration rate was calculated as liver weight/body weight x 100. Serum parameters were measured using a BS-200E Chemistry Analyzer (Mindray Medical international Ltd, Shenzhen, China). We determined the sample size for each experimental group based on our experience with similar studies, and is indicated in the figure legends.

### Precision cut liver slices

Precision-cute liver slices from patients with cirrhosis (n = 8), fibrotic mice (n = 6), or control mice (n = 5) were prepared as described by de Graaf *et al.*^26^ Briefly, liver tissue was punched with a disposable 6-mm biopsy puncher and was immersed in 4% Ultrapure low-melting agarose (Invitrogen, Waltham, MA, USA). Agarose-stabilized liver tissue was sliced into 250-μm-thick slices using a VT1000S vibrating blade microtome (Leica Biosystems, Nussloch, Germany), while being submerged in ice-cold Krebs-Henseleit buffer. Precision cut liver slices (PCLS) were pre-incubated in Williams medium E (Gibco, Waltham, MA, USA) supplemented with 2 mM L-glutamine, 50 U/ml penicillin/streptomycin at 37 °C in a 5% CO_2_ humidified incubator for 90 min to allow recovery after the cut. Then, four PCLS obtained from a single mouse or patient were placed on top of each MEEC or AM and co-cultured in direct contact in a 12-well plate for 24 h. At the end of the treatment, PCLS were carefully removed from the co-culture, gently separated from the underlying MEECs, washed twice with PBS, and then stored at −80 °C for ATP determination and RNA isolation. Cell viability was assessed in duplicate by measuring the content of ATP with the ATP Bioluminescence Assay Kit CLS II (Roche, Mannheim, Germany) as described in the supplementary data. The culture medium was collected to measure the levels of aspartate aminotransferase (AST) using a BS-200E Chemistry Analyzer (Mindray Medical International Ltd).

### Statistical analysis

Data are expressed as means ± SD. Results were analyzed by one-way ANOVA, the unpaired Student’s *t* test, or paired *t* test when appropriate (GraphPad Prism v8.0.1; GraphPad Software Inc., San Diego, CA, USA). Data were tested for assumptions before the use of these statistical tests. Differences were considered significant at *p* <0.05.

## Results

### MEECs protect hepatic viability and reduce inflammation in PCLS from patients with cirrhosis

The 3D conformation of the denatured collagen scaffold Gelfoam® was characterized by scanning electron microscopy (SEM) revealing a uniform, highly porous, fibrous structure with interconnected concentric pores (Fig. S1A). Human umbilical vein endothelial cells (HUVECs) were seeded into these 3D compressed collagen matrices and cultured for 2 weeks to attain stable cell numbers and a phenotype consistent with an optimized regulatory function. Calcein and Propidium Iodide staining confirmed EC viability (Fig. S1B and Video S1), and Wheat Germ Agglutinin (WGA) and Phalloidin staining revealed cell alignment within the matrices in a 3D elongated shape consistent with physiological integrity (Fig. S1C). ECs were able to migrate through the scaffold pores and channels and align in circular and tubular structures within the scaffold (Fig. S1C). These structures were reminiscent of vascular networks.

Supplementary data to this article can be found online at https://doi.org/10.1016/j.jhepr.2025.101617.

**Fig. 1:**
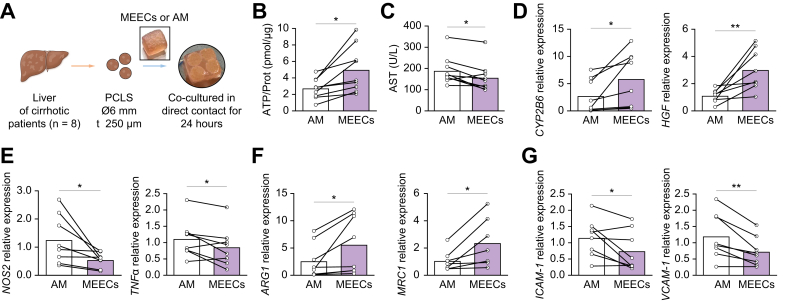
MEECs protect hepatic viability and reduce inflammation in PCLS from patients with cirrhosis. (A) Schematic of PCLS obtention and treatment time points. PCLS obtained from each patients with cirrhosis were divided into two groups; one group was co-cultured in direct contact with AM and the other with MEECs for 24 h. The effects of the treatment with AM on PCLS were compared with those on PCLS from the same patient. (B) ATP content of PCLS co-cultured in direct contact with AM or MEECs for 24 h (*p =* 0.0105). (C) AST levels in the culture medium of PCLS treated with AM or MEECs (*p =* 0.0179). (D) Hepatic expression of genes related to cell viability: *CYP2B6* (*p =* 0.0375, paired *t* test) and *HGF* (*p =* 0.0089) in PCLS treated with either AM or MEECs. (E–G) Hepatic expression of (E) the proinflammatory genes *NOS2* (*p =* 0.0362) and *TNFα* (*p =* 0.0488), (F) the anti-inflammatory genes *ARG1* (*p =* 0.0377) and *MRC1* (*p =* 0.0275), and (G) ICAM-1 (*p =* 0.0407) and VCAM-1 (*p =* 0.0054) in PCLS treated with either AM or MEECs. Data are presented as mean ± SD, n = 8 (D–G) or 9 (B,C) patients, analyzed by paired *t* test; ∗*p* <0.05 ∗∗ *p* <0.01. AM, acellular matrix; ARG1, arginase 1; AST, aspartate aminotransferase; CYP2B6, cytochrome P450 2B6; HGF, hepatocyte growth factor; ICAM-1, intercellular adhesion molecule 1; MEECs, matrix-embedded endothelial cells; MRC1, mannose receptor 1; NOS2, nitric oxide synthase 2; PCLS, precision cut liver slices; TNFα, tumor necrosis factor alpha; VCAM-1, vascular cell adhesion molecule 1.

The biological effects of MEECs on cirrhosis were evaluated on PCLS from liver biopsies of patients with cirrhosis (Fig.1 A). The PCLS model preserves cell–cell interactions within the original hepatic architecture and is a reliable tool to investigate liver tissue response to treatment.^27^ Eight patients (one female and seven males, 61.3 ± 7 years) with decompensated liver cirrhosis for 4.2 ± 4 years and model for end-stage liver disease (MELD) scores between 18 and 25 were selected from a single center (Hospital Clinic of Barcelona, Spain). Demographic and baseline characteristics of participants are shown in Table S1. Human cirrhotic PCLS (hPCLS) obtained from each patient were divided into two treatment groups; (1) co-culture of PCLS in direct contact with AM for 24 h; and (2) co-culture of PCLS with MEECs for 24 h. The effects of the treatment with AM on PCLS were compared with the effects on PCLS from the same patient treated with MEECs. The effects of MEECs on tissue viability of hPCLS were evaluated by measuring the tissue content of ATP, which is a sensitive marker for slice integrity and correlates with the number of cells that produce energy in tissue slice cultures.^26^^,^^28^ The ATP levels in hPCLS treated with MEECs were higher than in those treated with AM (Fig. 1B) and above the standard threshold for acceptable viability (2–12 nmol ATP per milligram of protein, as previously reported^26^). To further evaluate the viability of hPCLS, the levels of AST were measured in the culture medium. MEEC treatment reduced the release of AST into the medium, denoting a reduction in hepatocyte injury (Fig. 1C). This improved hepatocyte viability in hPCLS treated with MEECs correlated with an increase in the hepatic expression of the metabolic enzyme cytochrome P450 2B6 (CYP2B6) and hepatocyte growth factor (HGF), another marker of hepatocyte viability (Fig. 1D). Overall, these results suggest that MEECs treatment protects cell viability and reduces liver damage in hPCLS.

We further investigated whether the anti-inflammatory properties of MEECs^29^ could be of therapeutic interest for chronic liver injury. Expression of the proinflammatory genes encoding nitric oxide synthase (*NOS2*) and tumor necrosis factor alpha (*TNFα*) was lower in hPCLS treated with MEECs compared with those treated with AM (Fig. 1E). By contrast, the expression of the anti-inflammatory genes encoding arginase 1 (*ARG1*) and mannose receptor (*MRC1*) was increased in hPCLS co-cultured with MEECs (Fig. 1F), indicating mitigation of hepatic inflammation. MEECs decrease inflammation, in part, through the reduction of monocyte recruitment to inflamed endothelium.^29^ However, proinflammatory LSECs also have an essential role in immune cell recruitment and the expansion of liver inflammation through the expression of adhesion molecules.^30^ Expression of the genes encoding intercellular adhesion molecule 1 (*ICAM-1*) and vascular cell adhesion molecule 1 (*VCAM-1*) in hPCLS co-cultured with MEECs was significantly lower than in those co-cultured with AM (Fig.1G), suggesting amelioration of the inflammatory phenotype of LSECs in cirrhotic livers.

### MEEC treatment protects cell viability and reduces liver damage in PCLS from fibrotic and healthy mice

**Fig. 2:**
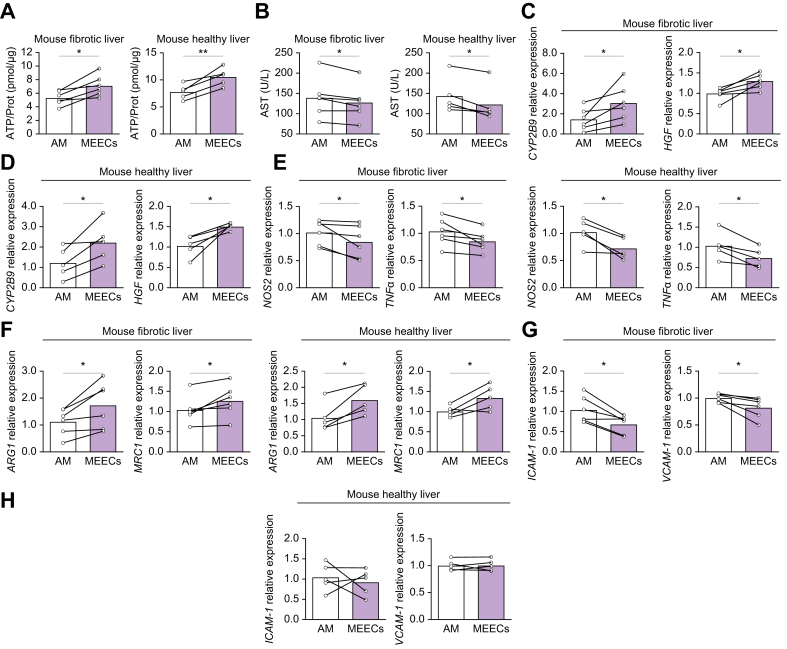
MEECs treatment protects hepatic viability and reduces inflammation in PCLS from fibrotic and healthy mice. (A) ATP content of PCLS from fibrotic (CH) and healthy mice (CT) co-cultured in direct contact with either AM or MEECs (CH, *p =* 0.0102; CT, *p =* 0.0205). (B) AST levels in the culture medium of fibrotic and healthy PCLS co-cultured with either AM or MEECs (CH, *p =* 0.0245; CT, *p =* 0.0184). (C) Hepatic expression of genes related to cell viability: *CYP2B9* (*p =* 0.0415) and *HGF* (*p =* 0.0121) in PCLS from fibrotic mice co-cultured with either AM or MEECs. (D) Hepatic expression of genes related to cell viability: *CYP2B9* (*p =* 0.0315) and *HGF* (*p =* 0.0178) in PCLS from healthy mice co-cultured with either AM or MEECs. (E) Hepatic expression of the proinflammatory genes *NOS2* and *TNFa* in PCLS from fibrotic (*NOS2*, *p* = 0.0499; *TNFα*, *p* = 0.0265) or healthy (*NOS2*, *p* = 0.0193; *TNFα*, *p* = 0.0144) mice treated with either AM or MEECs. (F) Hepatic expression of the anti-inflammatory genes *ARG1* and *MRC1* in PCLS from fibrotic (*ARG1*, *p* = 0.0250; *MRC1*, *p* = 0.0437) or healthy (*ARG1*, *p* = 0.0145; *MRC1*, *p* = 0.0422) mice treated with either AM or MEECs. (G) Hepatic expression of *ICAM-1* (*p* = 0.0125) and *VCAM-1* (*p* = 0.0235) in PCLS from fibrotic liver treated with either AM or MEECs. (H) Hepatic expression of *ICAM-1* (*p* = 0.6175) and *VCAM-1* (*p* = 0.9835) in PCLS from healthy mouse liver treated with either AM or MEECs. Data are presented as mean ± SD, n = 5 (CT) or 6 (CH) mice, analyzed by paired *t* test; ∗*p* <0.05 ∗∗*p* <0.01. AM, acellular matrix; ARG1, arginase 1; AST, aspartate aminotransferase; CYP2B6, cytochrome P450 2B6; HGF, hepatocyte growth factor; ICAM-1, intercellular adhesion molecule 1; MEECs, matrix-embedded endothelial cells; MRC1, mannose receptor 1; NOS2, nitric oxide synthase 2; PCLS, precision cut liver slices; TNFα, tumor necrosis factor alpha; VCAM-1, vascular cell adhesion molecule 1.

More precise evaluation of the effects of MEECs was achieved using PCLS from fibrotic and healthy mice. Fibrosis was induced by i.p. injection of CCl_4_ (twice a week for 8 weeks) in BALB/c mice. After 8 weeks, mice were euthanized and PCLS from fibrotic and healthy mice were co-cultured with MEECs for 24 h. As in the human samples, PCLS from fibrotic and healthy mice co-cultured with MEECs showed an increase in ATP content (Fig. 2A) and released less AST into the culture medium (Fig. 2B) compared with those treated with AM. Hepatic expression of *CYP2B9* and *HGF* was also higher in PCLS treated with MEECs than in those treated with AM (Fig. 2C,D). In line with the results in hPCLS, treatment with MEECs reduced inflammation in PCLS from fibrotic and healthy mice through the decrease in the expression of the proinflammatory genes *NOS2* and *TNFα* (Fig. 2E) and the increase in the expression of the anti-inflammatory genes *ARG1* and *MRC1* (Fig. 2F). The LSEC proinflammatory phenotype was also reduced in fibrotic PCLS co-cultured with MEECs compared with those co-cultured with AM, as evidenced by a decrease in the expression of *ICAM-1* and *VCAM-1* (Fig. 2G). Healthy PCLS did not show any changes in the expression of *ICAM-1* or *VCAM-1*, likely due to the absence of preexisting chronic liver damage (Fig. 2H).

### MEECs synthetize hepatoprotective and anti-inflammatory agents

**Fig. 3:**
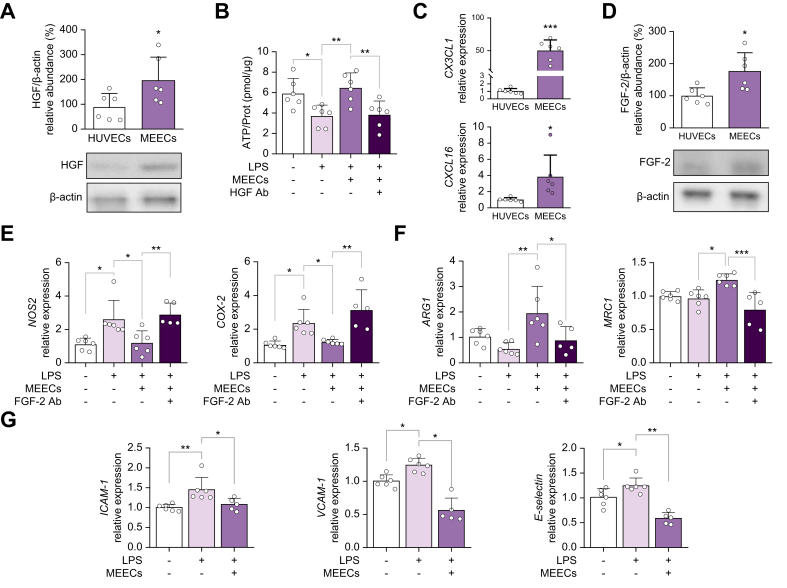
MEECs synthesize hepatoprotective and anti-inflammatory agents. (A) Western blot analysis of HGF and β-actin in 2D-ECs or MEECs, and relative HGF to β-actin protein abundance (%; *p* = 0.0391). (B) ATP levels of PCLS prestimulated with LPS and co-cultured with either MEECs^+^ or AM^-^ with or without HGF Ab (LPS^-^*vs.* LPS^+^, *p* = 0.0421; LPS^+^*vs.* LPS^+^ MEEC^+^, *p* = 0.00087; LPS^+^ MEEC^+^*vs.* LPS^+^ MEEC^+^ HGF Ab^+^, *p* = 0.0122). (C) Expression of the genes encoding the anti-inflammatory chemokines CX3CL1 (*p* <0.0001) and CXCL16 (*p* = 0.0249) in 2D-ECs and MEECs. (D) Western blot analysis of FGF-2 and β-actin in 2D-ECs and MEECs, and the relative FGF-2 to β-actin protein abundance (%; *p* = 0.0148). (E) Expression of the proinflammatory genes *NOS2* (LPS^-^*vs.* LPS^+^, *p* = 0.0144; LPS^+^*vs.* LPS^+^ MEEC^+^, *p* = 0.0221; LPS^+^ MEEC^+^*vs.* LPS^+^ MEEC^+^ FGF-2 Ab^+^, *p* = 0.0082) and *COX-2* (LPS^-^*vs.* LPS^+^, *p* = 0.0200; LPS^+^*vs.* LPS^+^ MEEC^+^, *p* = 0.0468; LPS^+^ MEEC^+^*vs.* LPS^+^ MEEC^+^ FGF-2 Ab^+^, *p* = 0.0011) in isolated hepatic CD11b^+^ macrophages prestimulated with LPS and treated with either MEECs^+^ or AM^-^ with or without FGF-2 Ab. (F) Gene expression of the anti-inflammatory genes *ARG1* (LPS^-^*vs.* LPS^+^, *p* = 0.5507; LPS^+^*vs.* LPS^+^ MEEC^+^, *p* = 0.0047; LPS^+^ MEEC^+^*vs.* LPS^+^ MEEC^+^ FGF-2 Ab^+^, *p* = 0.0472) and *MRC1* (LPS^-^*vs.* LPS^+^, *p* = 0.9756; LPS^+^*vs.* LPS^+^ MEEC^+^, *p* = 0.0157; LPS^+^ MEEC^+^*vs.* LPS^+^ MEEC^+^ FGF-2 Ab^+^, *p* = 0.0003) in isolated hepatic CD11b^+^ macrophages prestimulated with LPS and treated with either MEECs^+^ or AM^-^ with or without FGF-2 Ab. (G) Gene expression of *ICAM-1* (LPS^-^*vs.* LPS^+^, *p* = 0.0056; LPS^-^*vs.* LPS^+^ MEEC^+^, *p* = 0.8075; LPS^+^*vs.* LPS^+^ MEEC^+^, *p* = 0.0263), *VCAM-1* (LPS^-^*vs.* LPS^+^, *p* = 0.0169; LPS^-^*vs.* LPS^+^ MEEC^+^, *p* = 0.0002; LPS^+^*vs.* LPS^+^ MEEC^+^, *p* <0.0001), and *E-selectin* (LPS^-^*vs.* LPS^+^, *p* = 0.0497; LPS^-^*vs.* LPS^+^ MEEC^+^, *p* = 0.0011; LPS^+^*vs.* LPS^+^ MEEC^+^, *p* <0.0001) in hLSECs prestimulated with LPS and treated with either MEECs^+^ or AM^-^. Data are presented as mean ± SD, n = 6 (A,D) or experiments were performed in triplicates in two independent experiments (B,E–G), analyzed by Student’s *t* test (A,C,D) or one-way ANOVA (B,E–G); ∗*p* ≤0.05, ∗∗*p* ≤0.01, ∗∗∗*p* ≤0.001. 2D-ECs, endothelial cells in 2D cultures; Ab, antibodies; AM, acellular matrix; ARG1, arginase 1; COX-2, cyclooxygenase 2; EC, endothelial cell; FGF-2, fibroblast growth factor 2; HGF, hepatocyte growth factor; hLSECs, human liver sinusoidal endothelial cells; ICAM-1, intercellular adhesion molecule 1; LPS, lipopolysaccharide; MEECs, matrix-embedded endothelial cells; MRC1, mannose receptor 1; NOS2, nitric oxide synthase 2; VCAM-1, vascular cell adhesion molecule 1.

3D MEECs have a different transcriptome and secretome compared with ECs in 2D cultures (2D-ECs).^23^ We investigated which factors upregulated in 3D MEECs compared with 2D-ECs could explain the therapeutic and protective effects of MEECs in fibrotic and healthy livers, focusing on factors that were previously identified in MEECs secretome and transcriptome analyses.^23^ As anticipated, 3D MEECs showed higher levels of anti-apoptotic and mitogenic HGF compared with 2D-ECs (Fig. 3A).

To understand the protective role of MEECs on inflammation-induced injury, mouse PCLS were prestimulated with lipopolysaccharide (LPS) and then co-cultured with MEECs or AM. LPS reduced ATP content (cell viability marker) in PCLS, a fall that was fully rescued by MEECs treatment (Fig. 3B). The protective effects of MEECs on cell viability (ATP content) were completely blocked in the presence of a specific HGF antibody (Fig. 3B).

To understand the anti-inflammatory properties of MEECs, we investigated the expression of the anti-inflammatory chemokines CX3CL1 and CXCL16, and FGF-2 (a growth factor that promotes anti-inflammatory macrophage polarization^31^). MEECs exhibited higher expression levels of *CX3CL1*, *CXCL16* (Fig. 3C), and FGF-2 (Fig. 3D) compared with 2D-ECs. To explore the anti-inflammatory effects of MEECs on macrophages, murine isolated hepatic CD11b^+^ macrophages were prestimulated with LPS and then co-cultured with MEECs or AM. LPS markedly upregulated *NOS2* and cyclooxygenase (*COX-2*) expression in CD11b^+^ macrophages (Fig. 3E). These inflammatory effects were abolished when macrophages were treated with MEECs (Fig. 3E). MEECs also induced an increase in the expression of the anti-inflammatory genes *ARG1* and *MRC1* (Fig .3F), suggesting macrophage polarization to an anti-inflammatory phenotype. Anti-inflammatory macrophage polarization was completely blocked by antibodies against FGF-2 (Fig. 3E,F).

We then evaluated the ability of MEECs to modulate the inflammatory phenotype of LSEC. Human LSECs (hLSECs) were prestimulated with LPS to induce an inflammatory state and then co-cultured with MEECs or AM. LPS stimulation led to increased expression of the endothelial adhesion molecules *ICAM-1*, *VCAM-1*, and *E-selectin* (Fig. 3G). However, when inflamed hLSECs were co-cultured with MEECs, the expression of *ICAM-1*, *VCAM-1*, and *E-selectin* was significantly reduced (Fig. 3G). These findings indicate that MEECs can effectively reverse the proinflammatory phenotype of LSECs by downregulating the expression of key molecules involved in immune cell recruitment and inflammatory signaling.

### Perihepatic implantation of MEECs reduces liver injury and stimulates liver mass growth in mice with liver fibrosis

**Fig. 4:**
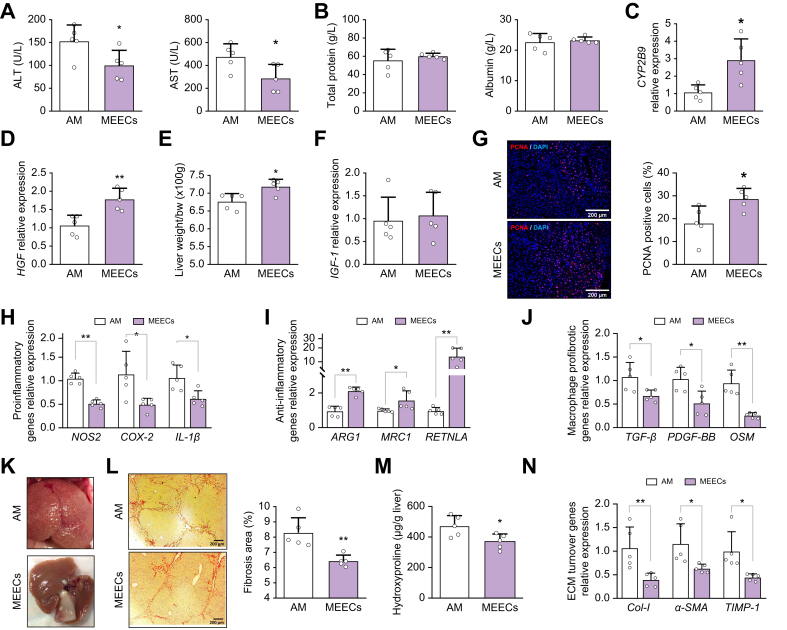
Beneficial effects of MEECs perihepatic implantation in fibrotic mice. (A,B) Serum levels of ALT (*p* = 0.0396), AST (*p* = 0.0338), albumin (*p* = 0.6648), and total protein (*p* = 0.4364) in fibrotic mice treated with perihepatic implants of either AM or MEECs for 1 week. (C,D) Hepatic expression of (C) *CYP2B9* (*p* = 0.0123) and (D) *HGF* (*p* = 0.0042) in fibrotic mice receiving either AM or MEEC perihepatic implants. (E–G) (E) Liver mass restoration rate (*p* = 0.0139), (F) hepatic expression of *IGF-1* (*p* = 0.7313), and (G) proliferating cell nuclear antigen immunofluorescence staining and percentage of PCNA-positive cells (*p* = 0.0271) in fibrotic mice treated with either AM or MEECs. (H) Hepatic expression of the proinflammatory genes encoding *NOS2* (*p* <0.0001), *COX-2* (*p* = 0.0212), and *IL-1β* (*p* = 0.0143) in fibrotic mice receiving perihepatic implants of either AM or MEEC. (I) Hepatic expression of the anti-inflammatory genes *ARG1* (*p* <0.0001), *MRC1* (*p* = 0.0437) and *RETNLA* (*p* = 0.0013) in fibrotic mice receiving either AM or MEEC perihepatic implants. (J) Hepatic expression of the macrophage-derived HSC activators *TGF-β* (*p* = 0.0261), *PDGF-BB* (*p* = 0.0118), and *OSM* (*p* = 0.0006) in fibrotic mice receiving either AM or MEEC perihepatic implants. (K) Macroscopic appearance of fibrotic liver after AM or MEEC treatment. (L,M) Sirius Red staining and quantification of (L) liver fibrosis area (*p* = 0.0045) and (M) hepatic hydroxyproline content (*p* = 0.0267) in fibrotic mice receiving either AM or MEEC perihepatic implants. (N) Hepatic expression of the ECM turnover genes encoding *Col-I* (*p* = 0.0119), *α-SMA* (*p* = 0.0263), and *TIMP-1* (*p* = 0.0193) in fibrotic mice receiving either AM or MEEC perihepatic implants. Data are presented as mean ± SD, *n* = 5 animals per group, analyzed by Student’s *t* test; ∗*p* ≤0.05, ∗∗*p* ≤0.01. α-SMA alpha-smooth muscle actin; ALT, alanine aminotransferase; AM, acellular matrix; ARG1, arginase 1; AST, aspartate aminotransferase; Col-I, collagen-I; COX-2, cyclooxygenase 2; CYP2B6, cytochrome P450 2B6; ECM, extracellular matrix; HGF, hepatocyte growth factor; HSC, hepatic stellate cell; IGF-1, insulin growth factor-1; MEEC, matrix-embedded endothelial cell; MRC1, mannose receptor 1; NOS2, nitric oxide synthase 2; OSM, oncostatin M; PCNA, Proliferating cell nuclear antigen; PDGF-BB, platelet-derived growth factor-BB; RETNLA, resistin-like alpha; TIMP-1, tissue inhibitor of metalloproteinases-1; TGF-β, transforming growth factor-beta.

To define whether the biological effects of MEECs could have sustained long-term therapeutic effects on liver fibrosis, we implanted MEECs or AM between the median and right lobe of the liver of fibrotic mice. Seven days after implantation, fibrotic mice treated with MEECs showed a significant reduction in hepatocyte damage compared with mice treated with AM, as evidenced by a reduction in the levels of serum transaminases (Fig. 4A) with no adverse effects on hepatic function, as evidenced by stable serum levels of albumin and total protein (Fig. 4B). The reduction in liver injury was accompanied by a significant improvement in hepatic viability, reflected by an increase in the hepatic expression of *CYP2B9* (Fig. 4C) and *HGF* (Fig. 4D). This increase in *HGF* expression in fibrotic mice implanted with MEECs was associated with a higher liver mass percentage (Fig. 4E). Insulin growth factor 1 (*IGF-1*), another common hepatocyte proliferation inducer, did not show significant changes (Fig. 4F). Proliferating cell nuclear antigen (PCNA) staining showed a higher number of proliferating cells in livers of mice implanted with MEECs (Fig. 4G) compared with mice receiving AM. These results suggest that the hepatic proliferation stimulated by the perihepatic implantation of MEECs was driven by HGF.

### Perihepatic implantation of MEECs reduces hepatic inflammation and fibrosis in mice with liver fibrosis

We investigated whether treatment with MEECs could also exert long-term anti-inflammatory effects in chronic liver injury in mice. Perihepatic implantation of MEECs into fibrotic mice significantly reduced the hepatic expression of proinflammatory genes (*NOS2*, *COX-2*, and *IL-1β*; Fig.4H), increased the hepatic expression of anti-inflammatory markers (*ARG1*, *MRC1* and resistin-like alpha [*RETNLA*]; Fig. 4I), and downregulated the hepatic expression of the endothelial adhesion molecules *ICAM-1* and *VCAM-1* (Fig. S2A). MEECs also promoted a significant reduction in the hepatic expression of the macrophage-derived HSC activators transforming growth factor-beta (*TGF-β*), platelet-derived growth factor-BB (*PDGF-BB*), and oncostatin M (*OSM*) (Fig.4J). These inhibitory effects on HSC activation were associated with hepatic ECM remodeling. Indeed, a change in the macroscopic aspect of fibrotic livers from micronodular to a less fibrotic liver appearance was visually appreciated after perihepatic implantation of MEECs (Fig. 4K). Histological analysis revealed that implantation of MEECs reduced porto-portal and porto-central bridging fibrosis compared with fibrotic livers implanted with AM, resulting in a 21% reduction in hepatic fibrosis area (Fig. 4L), along with a decrease in hepatic hydroxyproline content (Fig. 4M). Collagen-I (*Col-I*), alpha-smooth muscle actin (*α-SMA*), and tissue inhibitor of metalloproteinases-1 (*TIMP-1*) hepatic expression was also significantly reduced (Fig. 4N). Interestingly, MEECs treatment also enhanced the hepatic expression of matrix metalloproteinase (*MMP*)-9 and *MMP-2*, which actively participate in ECM degradation and fibrosis regression (Fig. S2B).

We then investigated possible antifibrotic agents that are synthesized at higher levels by 3D MEECs than by 2D-ECs. We found a significant increase in the expression of HS (a known inhibitor of HSC^15^) in MEECs compared with 2D-ECs (Fig. S3A). MEECs also showed a significant increase in *MMP-2* and *MMP-9* expression (Fig. S3B).

### Subcutaneous implantation of MEECs does not reduce inflammation or fibrosis in fibrotic mice

**Fig. 5:**
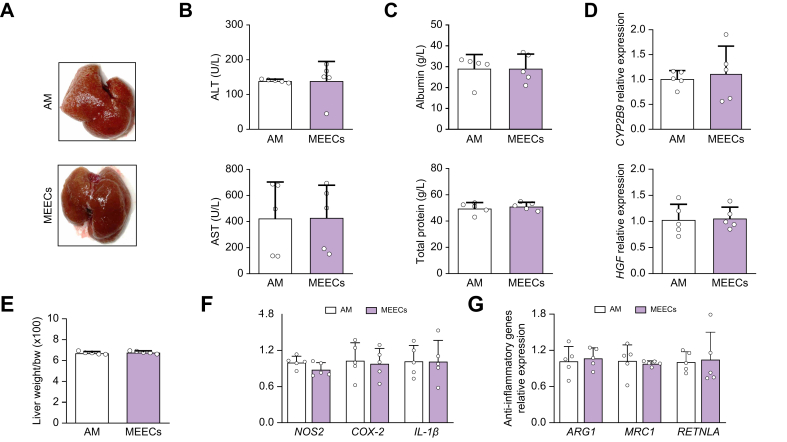
Subcutaneous implantation of MEECs does not reduce inflammation or fibrosis in fibrotic mice. (A) Macroscopic appearance of the fibrotic liver after subcutaneous treatment with either AM or MEECs for 1 week. (B,C) Serum levels of ALT (*p* = 0.9938), AST (*p* = 0.9790), albumin (*p* = 0.9964), and total protein (*p* = 0.5271) in fibrotic mice treated subcutaneously with either AM or MEECs for 1 week. (D) Hepatic expression of *CYP2B9* (*p* = 0.6887) and *HGF* (*p* = 0.8587) in fibrotic mice treated with either AM or MEECs. (E) Liver mass restoration in fibrotic mice treated subcutaneously with either AM or MEECs (*p* = 0.4353). (F) Hepatic expression of the proinflammatory genes *NOS2* (*p* = 0.1131) *COX-2* (*p* = 0.7696), and *IL-1β* (*p* = 0.9795) in fibrotic mice treated subcutaneously with either AM or MEECs. (G) Hepatic expression of the anti-inflammatory genes *ARG1* (*p* = 0.7179), *MRC1* (*p* = 0.6944), and *RETNLA* (*p* = 0.8488) in fibrotic mice treated subcutaneously with either AM or MEECs. Data are presented as mean ± SD, *n* = 5 animals per group, analyzed by Student’s *t* test; ∗*p* ≤0.05, ∗∗*p* ≤0.01. ALT, alanine aminotransferase; AM, acellular matrix; ARG1, arginase 1; AST, aspartate aminotransferase; COX-2, cyclooxygenase 2; CYP2B6, cytochrome P450 2B6; HGF, hepatocyte growth factor; MEEC, matrix-embedded endothelial cell; MRC1, mannose receptor 1; NOS2, nitric oxide synthase 2; RETNLA, resistin-like alpha.

To examine the dependency between the therapeutic effects of MEECs and the site of implantation, we studied whether subcutaneous MEEC implants could exert hepatic protective effects as a bioactive cellular patch. MEECs or AM were subcutaneously implanted for 7 days on the right upper quadrant of the abdomen of fibrotic mice. We did not observe macroscopic changes in the micronodular appearance between fibrotic livers implanted with AM or MEECs (Fig. 5A). Serum levels of transaminases, albumin, and total protein were not significantly different in animals treated subcutaneously with either MEECs or AM (Fig. 5B,C). Similarly, no changes in *CYP2B9* or *HGF* expression were observed after subcutaneous MEEC implantation (Fig. 5D) and neither were any effects found on liver mass restoration (Fig. 5E). As expected, no differences were found in the hepatic expression of proinflammatory (Fig. 5F), anti-inflammatory (Fig. 5G), and profibrotic (Fig. S4A) factors between fibrotic mice subcutaneously implanted with either MEECs or AM. Consequently, no changes were observed in the liver fibrosis area (Fig. S4B and C) and mRNA expression of *Col-I*, *α-SMA*, or *TIMP-1* (Fig. S4D). These results suggest that the crosstalk between MEECs and the hepatic environment is lost with subcutaneous implantation and that close contact of MEECs with the fibrotic liver is required to maintain high local concentration of secreted factors and provide therapeutic benefits.

### Perihepatic implantation of MEECs in fibrotic hepatectomized mice reduces liver injury, improves regeneration, and reduces inflammation and fibrosis

**Fig. 6:**
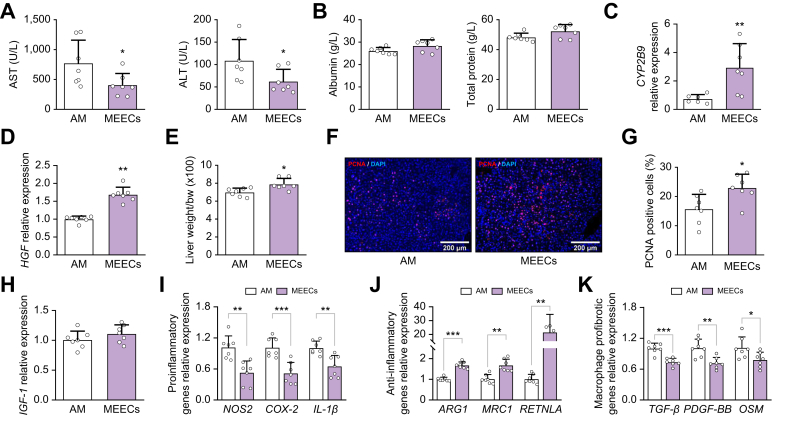
Beneficial effects of MEECs perihepatic implantation in fibrotic hepatectomized mice. (A,B) Serum levels of ALT (*p* = 0.0413), AST (*p* = 0.0438), albumin (*p* = 0.0701), and total protein (*p* = 0.0580) in fibrotic hepatectomized mice treated with perihepatic implants of either AM or MEECs for 1 week. (C,D) Hepatic expression of (C) *CYP2B9* (*p* = 0.0092) and (D) *HGF* (*p* <0.0001) in fibrotic hepatectomized mice receiving perihepatic implants of either AM or MEECs. (E–H) (E) Liver mass restoration rate (*p* = 0.0107), (F) PCNA immunofluorescence staining, (G) percentage of PCNA-positive cells (*p* = 0.0157), and (H) hepatic expression of *IGF-1* (*p* = 0.2186) in fibrotic hepatectomized mice treated with either AM or MEECs. (I) Hepatic expression of the proinflammatory genes encoding *NOS2* (*p* = 0.0014), *COX-2* (*p* = 0.0005), and *IL-1β* (*p* = 0.0024) in fibrotic hepatectomized mice treated with either AM or MEECs. (J) Hepatic expression of the anti-inflammatory genes *ARG1* (*p <*0.0001), *MRC1* (*p* = 0.0004), and *RETNLA* (*p* = 0.0012) in fibrotic hepatectomized mice treated with either AM or MEECs. (K) Hepatic expression of the macrophage-derived HSC activators *TGF-β* (*p* <0.0001), *PDGF-BB* (*p* = 0.0024), and *OSM* (*p* = 0.0192) in fibrotic hepatectomized mice treated with either AM or MEECs. Data are presented as mean ± SD, n = 7 animals per group, analyzed with Student’s *t* test; ∗*p* ≤0.05, ∗∗*p* ≤0.01, ∗∗∗*p* ≤0.001. ALT, alanine aminotransferase; AM, acellular matrix; ARG1, arginase 1; AST, aspartate aminotransferase; COX-2, cyclooxygenase 2; CYP2B6, cytochrome P450 2B6; HGF, hepatocyte growth factor; HSC, hepatic stellate cell; IGF-1, insulin growth factor-1; MEEC, matrix-embedded endothelial cell; MRC1, mannose receptor 1; NOS2, nitric oxide synthase 2; OSM, oncostatin M; PCNA, proliferating cell nuclear antigen; PDGF-BB, platelet-derived growth factor-BB; RETNLA, resistin-like alpha; TGF-β, transforming growth factor-beta.

To understand the potential therapeutic benefits of MEECs in the context of hepatic resection in a fibrotic liver (such as commonly occurs in clinics), we implanted MEECs perihepatically into mice with liver fibrosis and 40% hepatectomy. Treatment with MEECs reduced liver damage in fibrotic hepatectomized mice compared with animals receiving AM, as denoted by the reduction in serum transaminases levels (AST and alanine aminotransferase [ALT]) (Fig. 6A) without observing any changes in serum levels of albumin and total protein (Fig. 6B). MEECs promoted an increase in *CYP2B9* and *HGF* in the liver of fibrotic hepatectomized mice (Fig. 6C,D). The increase in *HGF* was accompanied by a significant rise in liver mass restoration (Fig. 6E). PCNA staining showed a higher number of proliferating cells in livers from mice implanted with MEECs (Fig. 6F) compared with animals receiving AM. Indeed, MEECs promoted a 45% increase in proliferative cells in hepatectomized fibrotic livers compared with animals implanted with AM (Fig. 6G). The hepatic proliferation stimulated by MEECs implantation was mainly attributed to the increase in *HGF* given that mice did not show an upregulation in *IGF-1* expression (Fig. 6H). MEECs promoted a reduction in the hepatic expression of macrophage proinflammatory genes (Fig. 6I), concomitant with an increased expression of macrophage anti-inflammatory genes (Fig. 6J), and a reduction in the hepatic expression of adhesion molecules involved in macrophage recruitment, such as *ICAM-1* and *VCAM-1* (Fig. S5A). This macrophage phenotype switch was accompanied by a reduction in the expression of macrophage-derived signals involved in HSC activation (Fig. 6K). This was associated with a significant reduction in liver fibrosis (Fig. S5B) and mRNA expression of *Col-I*, *α-SMA*, and *TIMP-1* (Fig. S5C). MEECs also significantly induced the expression of hepatic *MMP-9* and *MMP-2* (Fig. S5D). Interestingly, we also observed that MEECs were able to sense the hepatic milieu of injured liver after hepatectomy and adapt their phenotype: MEECs expressed the LSEC-specific marker CD32b at the implant–liver interface (Fig. S6), indicating that they remain responsive to the environment. Indeed, it has been previously described that MEECs acquire an endothelial progenitor-like cell (EPC) phenotype and can switch to different endothelial subsets.^23^

## Discussion

This study has defined novel concepts for the protective role of ECs in liver homeostasis. MEECs allow for the dosing of healthy human ECs to regulate liver inflammation and restore hepatic integrity. Indeed, human MEECs assist the restoration of hepatic perfusion and prevention of hepatocyte apoptosis in healthy mice undergoing partial hepatectomy.^22^ Here, we show that the protection promoted by MEECs, and their therapeutic effects can be extended to chronic liver injury, characterized by hepatocyte apoptosis, inflammation, and fibrosis. Previous data on MEEC resistance to inflammatory factors^24^ and even to uremic milieu^25^ led us to believe that MEECs could remain active even within the hepatic milieu in chronic liver disease and exert their beneficial properties to treat liver inflammation. Our results indicate that MEECs preserve hepatic viability in PCLS from patients with cirrhosis and fibrotic and healthy mice. MEECs treatment displayed not only protective effects on the viability of hepatic cells, but also anti-inflammatory effects in PCLS by inducing macrophage polarization to an anti-inflammatory phenotype. MEECs also ameliorated the LSEC inflammatory and dysfunctional phenotype in PCLS from patients with cirrhosis and fibrotic mice. We found that MEECs release high amounts of HGF (a known hepatocyte cytoprotective and anti-apoptotic factor in chronic liver injury^32^^,^^33^). This explains, in part, how MEECs preserve hepatocyte viability and reduce damage in human cirrhotic and mouse fibrotic or healthy PCLS. MEECs treatment increased the ATP content and *CYP2B6* expression, suggesting an increase in energy production, cell metabolism, and detoxification. Indeed, some studies have described the role of HGF in the protection of mitochondrial physiology via mTOR signaling^34^ and the restoration of mitochondrial and endoplasmic reticular homeostasis.^35^ The nature of these dynamic and unique hepatoprotective paracrine properties of MEECs reveals much about native hepatic pathophysiology and repair, and opens new avenues for their potential use in liver transplantation either in the protection of the liver recipient or in the storage and preservation of liver graft donors. Although organ transplantation is the most effective therapy for advanced liver disease,^3^ the shortage of donor livers considered suitable for transplantation and transplant logistics have driven the need for additional methods for organ preservation and reconditioning.^36^ This novel approach could improve donor organ quality and, subsequently, patient morbidity and survival after transplantation.

It is well known that ECs control the recruitment of monocytes and the behavior of macrophages after liver injury.^37^^,^^38^ We showed that MEECs secrete CXCL16 and CX3CL1, cytokines associated with anti-inflammatory macrophage infiltration and M2-like anti-inflammatory macrophage polarization.^39^^,^^40^ Moreover, MEECs secrete high amounts of FGF-2, which promotes anti-inflammatory macrophage polarization, in line with other investigations.^31^ LSECs also have an essential role in the control of liver inflammation in chronic liver disease through upregulation of cell adhesion molecules and recruitment of immune cells.^30^ MEECs ameliorated the dysfunctional and inflammatory phenotype of LSEC by downregulating the expression of *ICAM-1*, *VCAM-1*, and *E-selectin*, confirming previous evidence for MEECs reducing the recruitment and infiltration of immune cells to inflamed tissues.^29^^,^^41^ These findings could explain, in part, the anti-inflammatory properties of MEECs in fibrotic livers. The need for proximity of this cell therapy reflects the power of local paracrine regulation above and beyond an endocrine-like effect. Subcutaneous implantation of MEECs failed to display therapeutic effects on liver inflammation, regeneration, or fibrosis, highlighting the major importance of close contact between MEECs and the injured tissue to exhibit protective properties. MEECs require dynamic communication and adaptation to the adjacent injured cells. For this reason, MEECs implants might be especially indicated for donor and recipient organs involved in liver transplantation.

Our data revealed that HS secreted by MEECs might directly mitigate HSC activation and fibrogenesis. Analogous beneficial outcomes reducing myofibroblast proliferation and intimal hyperplasia have been described with perivascular placement of MEECs implants containing allogeneic aortic ECs on injured carotid arteries.^42^ Moreover, we showed that MEECs also inhibit HSC activation via downregulation of macrophage-derived profibrotic factors, such as *TGF-β*, *PDGF-BB*, and *OSM*, likely as a result of the influence that FGF-2 released by MEECs exerts on the macrophage phenotype. Previous investigations point out that FGF-2 exhibits antifibrotic properties in chronic liver disease.^15^^,^^43^ Indeed, treatment with FGF-2 was demonstrated to reduce HSC activation and collagen expression followed by attenuated liver fibrosis.^43^ Here, MEECs decreased HSC activation in fibrotic mouse livers, as evidenced by a marked reduction in hepatic expression of *Col-I*, *α-SMA*, and *TIMP-1*. Moreover, our data revealed that MEECs contribute to ECM degradation via upregulation of *MMP-2* and *MMP-9*. Overall, our findings support the potential of MEECs as a cell-based therapy for reducing liver fibrosis in the context of long-term treatment for chronic liver disease. Hepatic scars physically and spatially limit hepatocyte expansion to regenerate damaged areas. The reduction in liver fibrosis in conjunction with the effects of HGF secreted by MEECs could explain, in part, the stimulation of liver growth observed in hepatectomized fibrotic mice treated with MEECs.

In conclusion, perihepatic human MEEC implants preserve viability in fibrotic, cirrhotic, or healthy liver tissue and display anti-inflammatory properties. The protective properties of human MEECs on healthy liver point to a potential application of this cell therapy as a feasible *ex situ* preservation method for donor liver grafts in a sustainable manner with the use of commercially available HUVECs, avoiding the isolation of host LSECs or other EC types. Prolonged treatment with MEECs shows additional pro-regenerative and antifibrotic properties in mice with liver fibrosis. MEECs exert myriad beneficial effects for the treatment of chronic liver disease: (1) hepatoprotection and regeneration mediated, in part, by HGF production; (2) anti-inflammatory activity stimulated by the release of FGF-2 and CXCL16 and CX3CL1 chemokines; and (3) antifibrotic properties mainly orchestrated by HS and the ECM degradation enzymes MMP-2 and MMP-9. Future work will explore additional engineered EC-based therapies to decipher the multifaceted benefits of endothelial health in reducing hepatic injury, inflammation, and fibrosis, and enhancing liver repair.

## Abbreviations

2D-ECs, endothelial cells in 2D cultures; α-SMA, alpha-smooth muscle actin; Ab, antibodies; ALT, alanine aminotransferase; AM, acellular matrices; ARG1, arginase 1; ARRIVE, Animal Research: Reporting of In Vivo Experiments; AST, aspartate aminotransferase; CCl4, carbon tetrachloride; Col-I, collagen-I; COX-2, cyclooxygenase 2; CYP2B6, cytochrome P450 2B6; ECM, extracellular matrix; ECs, endothelial cells; EPC, endothelial progenitor-like cell; FGF-2, fibroblast growth factor 2; HGF, hepatocyte growth factor; hLSECs, human liver sinusoidal endothelial cells; hPCLS, human cirrhotic precision cut liver slices; HS, heparan sulfate; HSC, hepatic stellate cells; HUVECs, human umbilical vein endothelial cells; ICAM-1, Intercellular adhesion molecule 1; IGF-1, insulin growth factor 1; LPS, lipopolysaccharide; LSEC, liver sinusoidal endothelial cells; MEECs, matrix-embedded endothelial cells; MELD, model for end-stage liver disease; MHC, major histocompatibility complex; MMP, metalloproteinase; MRC1, mannose receptor 1; NOS2, nitric oxide synthase 2; OSM, oncostatin M; PCLS, precision cut liver slices; PCNA, proliferating cell nuclear antigen; PDGF-BB, platelet-derived growth factor-BB; RETNLA, resistin-like alpha; SEM, scanning electron microscope; SMC, smooth muscle cells; TGF-β, transforming growth factor-beta; Th, T helper; TIMP-1, tissue inhibitor of metalloproteinases-1; TNF-α, tumor necrosis factor alpha; VCAM-1, vascular cell adhesion molecule 1; WGA, Wheat Germ Agglutinin.

## Financial support

This work was supported by grants from the Spanish Ministerio de Ciencia, Innovación y Universidades (RTI2018-094734-B-C21 and PID2021-123426OB-I00, cofunded by 10.13039/501100004837MCIN/AEI/10.13039/501100011033 and by “10.13039/501100008530ERDF A way of making Europe”) to PM-L and WJ. PM-L. was additionally supported by a consolidation grant (CNS2022-135148 funded by 10.13039/501100004837MCIN/AEI/10.13039/501100011033 and 10.13039/501100000780European Union NextGeneration EU/PRTR) and a fellowship from the Ramon y Cajal Program (RYC2018-0Z23971-I) funded by the Spanish Ministerio de Ciencia e Innovación funded by 10.13039/501100004837MCIN/AEI/10.13039/501100011033 and FSE Invierte en tu Futuro. PM-L was also granted a fellowship from the 10.13039/501100003030AGAUR Beatriu de Pinós Program 2016 (BP-00236) of 10.13039/501100002809Generalitat de Catalunya and a Sheila Sherlock Research Fellowship from the 10.13039/501100009253EASL. This work was also supported by a grant from the Spanish Ministerio de Ciencia, Innovación y Universidades (PID2022-138243OB-I00 funded by 10.13039/501100004837MCIN/AEI/10.13039/501100011033 and by ‘10.13039/501100008530ERDF A way of making Europe’) to MM-R. MM-B and BS-C received a Formación de Profesorado Universitario (10.13039/100019478FPU) grant from the Spanish Ministerio de Ciencia, Innovación y Universidades and FSE Invierte en tu Futuro (Reference: FPU19/03323 and FPU22/02178). AM-L received a Formación de Personal Investigador grant from the Spanish Ministerio de Ciencia, Innovación y Universidades (Reference: PRE2019-088097). ERE was supported by a grant (R01 HL161069) from the 10.13039/100000002National Institutes of Health. The Centro de Investigación Biomédica en Red de Enfermedades Hepáticas y Digestivas (CIBERehd) is funded by the 10.13039/501100004587Instituto de Salud Carlos III. RedFibro (RED2022-134485-T) of the 2022 call for aid to ‘RESEARCH NETWORKS’, within the framework of the Programa Estatal del Plan Estatal de Investigación Científica, Técnica y de Innovación 2021–2023, Consolidated Research Group of the Generalitat de Catalunya AGAUR (2021 SGR 00881).

## Authors’ contributions

Conceived the study: PM-L, ERE. Acquired and analyzed the data: MM-B, AM-L, BS-C, MP, LM-M, PM-L. Obtained the human liver samples: DS-P, YF, FJS. Co-wrote the manuscript: MM-B, PM-L. Edited and reviewed the manuscript: MM-R, ERE, WJ. Approved the final version of the manuscript: all authors.

## Data availability

All data are available in the main text or the supplementary materials.

## Conflicts of interest

The authors declare no conflicts of interest that pertain to this work.

Please refer to the accompanying ICMJE disclosure forms for further details.

## References

[1] Asrani S.K., Devarbhavi H., Eaton J. (2019). Burden of liver diseases in the world. J Hepatol.

[2] Trautwein C., Friedman S.L., Schuppan D. (2015). Hepatic fibrosis: concept to treatment. J Hepatol.

[3] Jing L., Yao L., Zhao M. (2018). Organ preservation: from the past to the future. Acta Pharmacol Sin.

[4] Medrano-Bosch M., Moreno-Lanceta A., Melgar-Lesmes P. (2021). Nanoparticles to target and treat macrophages: the Ockham’s concept?. Pharmaceutics.

[5] Moreno-Lanceta A., Medrano-Bosch M., Melgar-Lesmes P. (2020). Single-walled carbon nanohorns as promising nanotube-derived delivery systems to treat cancer. Pharmaceutics.

[6] Moreno-Lanceta A., Medrano-Bosch M., Fundora Y. (2023). RNF41 orchestrates macrophage-driven fibrosis resolution and hepatic regeneration. Sci Transl Med.

[7] Gracia-Sancho J., Caparrós E., Fernández-Iglesias A. (2021). Role of liver sinusoidal endothelial cells in liver diseases. Nat Rev Gastroenterol Hepatol.

[8] Nugent M.A., Nugent H.M., Iozzo R.V. (2000). Perlecan is required to inhibit thrombosis after deep vascular injury and contributes to endothelial cell-mediated inhibition of intimal hyperplasia. Proc Natl Acad Sci U S A.

[9] Shao Y., Saredy J., Yang W.Y. (2020). Vascular endothelial cells and innate immunity. Arterioscler Thromb Vasc Biol.

[10] Poisson J., Lemoinne S., Boulanger C. (2017). Liver sinusoidal endothelial cells: physiology and role in liver diseases. J Hepatol.

[11] Amersfoort J., Eelen G., Carmeliet P. (2022). Immunomodulation by endothelial cells — partnering up with the immune system?. Nat Rev Immunol.

[12] Peralta C., Jiménez-Castro M.B., Gracia-Sancho J. (2013). Hepatic ischemia and reperfusion injury: effects on the liver sinusoidal milieu. J Hepatol.

[13] Verhulst S., van Os E.A., De Smet V. (2021). Gene signatures detect damaged liver sinusoidal endothelial cells in chronic liver diseases. Front Med.

[14] Methe H., Nanasato M., Spognardi A.M. (2010). T-helper 2 cells are essential for modulation of vascular repair by allogeneic endothelial cells. J Heart Lung Transpl.

[15] Seitz T., Hellerbrand C. (2021). Role of fibroblast growth factor signalling in hepatic fibrosis. Liver Int.

[16] Ishikawa T., Terai S., Urata Y. (2007). Administration of fibroblast growth factor 2 in combination with bone marrow transplantation synergistically improves carbon-tetrachloride-induced liver fibrosis in mice. Cell Tissue Res.

[17] Liu F., Liu Z. Da, Wu N. (2009). Transplanted endothelial progenitor cells ameliorate carbon tetrachloride-induced liver cirrhosis in rats. Liver Transpl.

[18] Methe H., Hess S., Edelman E.R. (2008). The effect of three-dimensional matrix-embedding of endothelial cells on the humoral and cellular immune response. Semin Immunol.

[19] Methe H., Groothuis A., Sayegh M.H. (2007). Matrix adherence of endothelial cells attenuates immune reactivity: induction of hyporesponsiveness in allo- and xenogeneic models. FASEB J.

[20] Nickmann M., Saemisch M., Wilbert-Lampen U. (2013). Cell matrix contact modifies endothelial major histocompatibility complex class II expression in high-glucose environment. Am J Physiol Heart Circ Physiol.

[21] Methe H., Nugent H.M., Groothuis A. (2005). Matrix embedding alters the immune response against endothelial cells in vitro and in vivo. Circulation.

[22] Melgar-Lesmes P., Balcells M., Edelman E.R. (2017). Implantation of healthy matrix-embedded endothelial cells rescues dysfunctional endothelium and ischaemic tissue in liver engraftment. Gut.

[23] Abraham E., Gadish O., Franses J.W. (2017). Matrix-embedded endothelial cells attain a progenitor-like phenotype. Adv Biosyst.

[24] Saemisch M., Nickmann M., Riesinger L. (2018). 3D matrix-embedding inhibits cycloheximide-mediated sensitization to TNF-alpha-induced apoptosis of human endothelial cells. J Tissue Eng Regen Med.

[25] Chitalia V.C., Murikipudi S., Indolfi L. (2011). Matrix-embedded endothelial cells are protected from the uremic milieu. Nephrol Dial Transpl.

[26] De Graaf I.A.M., Olinga P., De Jager M.H. (2010). Preparation and incubation of precision-cut liver and intestinal slices for application in drug metabolism and toxicity studies. Nat Protoc.

[27] Simon E., Motyka M., Prins G.H. (2023). Transcriptomic profiling of induced steatosis in human and mouse precision-cut liver slices. Sci Data.

[28] Dewyse L., Reynaert H., van Grunsven L.A. (2021). Best practices and progress in precision-cut liver slice cultures. Int J Mol Sci.

[29] Indolfi L., Baker A.B., Edelman E.R. (2012). The role of scaffold microarchitecture in engineering endothelial cell immunomodulation. Biomaterials.

[30] McConnell M.J., Kostallari E., Ibrahim S.H. (2023). The evolving role of liver sinusoidal endothelial cells in liver health and disease. Hepatology.

[31] Im J.H., Buzzelli J.N., Jones K. (2020). FGF2 alters macrophage polarization, tumour immunity and growth and can be targeted during radiotherapy. Nat Commun.

[32] Rizvi F., Everton E., Smith A.R. (2021). Murine liver repair via transient activation of regenerative pathways in hepatocytes using lipid nanoparticle-complexed nucleoside-modified mRNA. Nat Commun.

[33] Yu Y., Yao A.-H., Chen N. (2007). Mesenchymal stem cells over-expressing hepatocyte growth factor improve small-for-size liver grafts regeneration. Mol Ther.

[34] Peng F., Chang W., Sun Q. (2020). HGF alleviates septic endothelial injury by inhibiting pyroptosis via the mTOR signalling pathway. Respir Res.

[35] Zineldeen D.H., Tahoon N.M., Sarhan N.I. (2023). AICAR ameliorates non-alcoholic fatty liver disease via modulation of the HGF/NF-κB/SNARK signaling pathway and restores mitochondrial and endoplasmic reticular impairments in high-fat diet-fed rats. Int J Mol Sci.

[36] Ceresa C.D.L., Nasralla D., Pollok J.-M. (2022). Machine perfusion of the liver: applications in transplantation and beyond. Nat Rev Gastroenterol Hepatol.

[37] Medrano-Bosch M., Simón-Codina B., Jiménez W. (2023). Monocyte-endothelial cell interactions in vascular and tissue remodeling. Front Immunol.

[38] Melgar-Lesmes P., Edelman E.R. (2015). Monocyte-endothelial cell interactions in the regulation of vascular sprouting and liver regeneration in mouse. J Hepatol.

[39] Wang X.-Q., Zhou W.-J., Hou X.-X. (2018). Trophoblast-derived CXCL16 induces M2 macrophage polarization that in turn inactivates NK cells at the maternal–fetal interface. Cell Mol Immunol.

[40] Karlmark K.R., Zimmermann H.W., Roderburg C. (2010). The fractalkine receptor CX3CR1 protects against liver fibrosis by controlling differentiation and survival of infiltrating hepatic monocytes. Hepatology.

[41] Hess S., Methe H., Kim J.-O. (2009). NF-κB activity in endothelial cells is modulated by cell substratum interactions and influences chemokine-mediated adhesion of natural killer cells. Cell Transpl.

[42] Unterman S., Freiman A., Beckerman M. (2015). Tuning of collagen scaffold properties modulates embedded endothelial cell regulatory phenotype in repair of vascular injuries in vivo. Adv Healthc Mater.

[43] Sato-Matsubara M., Matsubara T., Daikoku A. (2017). Fibroblast growth factor 2 (FGF2) regulates cytoglobin expression and activation of human hepatic stellate cells via JNK signaling. J Biol Chem.

