# Supplementary material for: 3D endothelial cell scaffolds protect liver explants and exhibit therapeutic effects on liver fibrosis
Source: JHEP Rep. 2025 Oct 4;8(1):101617. doi: 10.1016/j.jhepr.2025.101617 (PMC12890448; doi:10.1016/j.jhepr.2025.101617)
Supplement: Multimedia component 1 [file mmc1.pdf]

3D endothelial cell scaffolds protect liver explants and exhibit therapeutic effects on liver fibrosis

Mireia Medrano-Bosch, Alazne Moreno-Lanceta, Blanca Simón-Codina, David Saavedra-Pérez, Yiliam Fundora, Francisco J Sánchez, Meritxell Perramón, Laura Macias-Muñoz, Manuel Morales-Ruiz, Elazer R. Edelman, Wladimiro Jiménez, Pedro Melgar-Lesmes

Table of contents

Supplementary materials and methods…………………………………………..2

Supplementary figures…………………………………………………………11

Supplementary table……………………………………………………………17

Supplementary video………………………………………………......separate file

Supplementary materials and methods

Cell culture conditions and seeding of MEECs

Human Umbilical Vein Endothelial Cells (HUVECs, ATCC) were grown in endothelial cell growth medium (EGM-2, Lonza): endothelial basal medium (EBM-2) supplemented with EGM-2 SingleQuots® Supplements, 10% of fetal bovine serum (FBS, Invitrogen), 2 mM L-Glutamine, and 50 U/mL penicillin/streptomycin (Gibco). HUVECs were cultured on gelatin-coated flasks (0.1% gelatin type A, Sigma) and passaged when they reached 80% confluence. For all the experiments, HUVECs were used at passages 2 to 5. For cell-matrix seeding, compressed denatured collagen matrices (Gelfoam, Pfizer, New York, NY) were cut into 1 x 1 x 0.3 cm blocks and hydrated in culture medium at 37ºC for 2h. Then 4.5 x 10^4 ECs (suspended in 50 µL media) were seeded onto one surface of the hydrated matrix and allowed to attach for 1.5h. Afterwards, the matrix was turned over and additional 4.5 x 10^4 ECs were added to infiltrate from the second side. After an additional 1.5h incubation period to enable cell attachment, each cell-seeded construct was carefully transferred to a separate 50 mL polypropylene tube containing 10 mL of culture medium. Matrices were cultured for 2 weeks under standard culture conditions (37ºC, 5% CO_2, humidified environment), with medium changes every 48 hours.

The compressed denatured 3D collagen matrices and MEECs were characterized by Scanning electron microscope (SEM) and confocal microscopy, respectively, as described in the supplementary materials and methods. The expression patterns of HUVECs and MEECs were compared by Western Blot and Real-time PCR. *In vitro* studies were designed to explore the effects of MEECs secretome on cell viability, macrophage polarization and LSEC phenotype. For cell culture experiments, a minimum of three experimental replicates were performed (the exact number of replicates is provided in the figure legends).

Characterization of compressed denatured 3D collagen matrices and MEECs by Scanning electron microscopy (SEM) and confocal microscopy

Scanning electron microscopy was used for the characterization of the porous structure of compressed denatured 3D collagen matrices. Samples were mounted on microscope holders with carbon tape and covered with a gold thin film to improve their electrical conductivity. The samples were observed with a Jeal JSM-7001F (Joel, Japan) operated at 15 kV on the TEM-SEM Electron Microscopy Unit from Scientific and Technological Centers (CCiTUB), Universitat de Barcelona.

HUVECs viability in 3D collagen matrices was characterized by co-staining MEECs with Calcein AM (1µM, Sigma-Aldrich, ref: 206700), Propidium Iodide (3µM, Sigma-Aldrich, ref: 537059) and Hoechst 33342 (1µM, Invitrogen, ref: H3570) for 30 minutes at 37°C in a 5% CO2 incubator. Then, MEECs were observed with a confocal microscope Leica TC5 SP5 on the Advanced Optical Microscopy Unit from Scientific and Technological Centers (CCiTUB), Universitat de Barcelona.

To evaluate the disposition and alignment of HUVECs within the 3D collagen matrices, MEECs were fixed with 10% buffered formaldehyde solution for 20 minutes at room temperature. Following fixation, MEECs were washed three times with phosphate-buffered saline (PBS) and stained with Wheat Germ Agglutinin (1.5 µL/mL, Thermo Scientific, ref: W11261) for 8 minutes under gentle shaking at room temperature. MEECs were then permeabilized with 0,1% Triton X-100 for 5 minutes, and stained with phalloidin (1.5µL/mL, Fisher Scientific, ref: A30106) and Hoechst 33342 (1 µM, Invitrogen, ref: H3570) for 30 minutes under gentle shaking at room temperature. Then, MEECs were observed with a confocal microscope Leica TC5 SP5 on the Advanced Optical Microscopy Unit from Scientific and Technological Centers (CCiTUB), Universitat de Barcelona.

ATP determination

ATP levels were measured following the manufacturer's protocol (ATP Bioluminescence Assay Kit CLS II, Roche). Briefly, PCLS were homogenized in Sonification solution using a Polytron PT 1200E (Polytronix Inc, Richardson, Texas), and the homogenate was centrifuged at 13,000 x g for 5 min. The supernatant was used for ATP measurement, and the pellet for protein quantification. For the ATP assay, 5 µL of the supernatant were diluted 10 times in 100 mM Tris–HCl, 2 mM EDTA buffer (pH 7.8) in a 96-well white plate. Then, 50 µL of luciferase reagent were added to each well, and luminescence was measured using a TECAN SPARK microplate reader (Tecan Group, Mannedorf, Switzerland). ATP levels were calculated from a log-log plot of the standard curve data and normalized to the total protein content of each slice, which was determined using the Pierce BCA Protein Assay Kit (Thermo Fisher Scientific, Basingstoke, UK).

Viability assay in precision cut liver slices stimulated with LPS and treated with MEECs in the presence of a specific antibody against HGF

For PCLS preparation, the inferior vena cava of anesthetized healthy mice was cut off and blood was allowed to drain for 1 min. PCLS from healthy mice were individually placed in 12-well plates in Williams medium E with 10% inactivated FBS, 2 mM L-Glutamine, and 50 U/mL penicillin/streptomycin. PCLS were pre-incubated for 120 min at 37°C in a 5% CO_2 incubator to allow the recovery after the cut. PCLS were then incubated with LPS (100 µg/mL, Sigma-Aldrich) or vehicle for 18 hours. Next, PCLS were transferred to 12-well plates in groups of three and were placed on top of each MEECs or AM for 32 hours. In a separate experimental condition, rabbit anti-HGF polyclonal antibodies (1:1000, Abcam, ref: ab24865) were added to the media of PCLS treated with MEECs to evaluate its involvement in PCLS viability and integrity. At the end of the treatment, cell viability was assessed in triplicates by measuring the content of

ATP with the ATP Bioluminescence Assay Kit CLS II (Roche, Mannheim, Germany). Experiments were performed in triplicates in two independent experiments and reported as mean ± SD.

Inflammation assay in isolated primary hepatic CD11b$^+$ macrophages treated with MEECs

Freshly isolated primary hepatic CD11b$^+$ macrophages were obtained from livers of healthy mice (control). Livers were collected from healthy control mice following euthanasia and transcardiac perfusion with a solution containing collagenase A (Roche Diagnostics). Livers were excised, minced, and digested in pre-warmed digestion buffer. Hepatocytes were removed by low-speed centrifugation (70 x g for 1 minute), while non-parenchymal cells were further purified using a 30% Histodenz gradient (Sigma-Aldrich). Macrophages were then isolated using anti-CD11b magnetic beads (Miltenyi Biotec, reference: 130-049-601). Freshly isolated primary hepatic CD11b$^+$ macrophages were seeded in 6-well plates at a cell density of 4×10^5 cells per well in complete Dulbecco's Modified Eagle's Medium (DMEM) supplemented with 10% inactivated fetal bovine serum (FBS), 2 mM L-Glutamine, 50 U/mL penicillin, and streptomycin. Then, macrophages were incubated with lipopolysaccharide (LPS, 100 ng/mL, Sigma-Aldrich) or vehicle for 16 hours. After stimulation, media was removed, and macrophages were co-cultured in indirect contact via inserts with MEECs or AM for 48 hours. CellQART® 6-well inserts (0.4 µm pore size and PET-membrane) containing MEECs or AM were placed on top of the wells containing isolated macrophages. In another condition, rabbit anti-FGF2 polyclonal antibody (1:1000, Thermo Fisher, ref: PA5-116495) was added to the insert media containing MEECs to evaluate its role in macrophage polarization. Following co-culture, macrophages were resuspended in TRIzol reagent (Invitrogen) for RNA isolation and analysis of pro-inflammatory and anti-inflammatory gene expression

by Real-Time PCR. Experiments were performed in triplicates in two independent experiments and reported as mean ± SD.

Inflammation assay in hLSEC treated with MEECs

Human LSEC (Innoprot, ref: P10652) were grown in endothelial cell growth medium (EGM-2, Lonza): endothelial basal medium (EBM-2) supplemented with EGM-2 SingleQuots® Supplements, 10% of fetal bovine serum (FBS, Invitrogen), 2 mM L-Glutamine, and 50 U/mL penicillin/streptomycin (Gibco). LSECs were cultured on fibronectin-coated flasks (Bovine plasma fibronectin, Invitrogen). LSECs were seeded in 6-well plates at a cell density of $1{,}5 \times 10^5$ cells per well. LSEC were incubated with lipopolysaccharide (LPS, 100 ng/mL, Sigma-Aldrich) or vehicle for 16 hours. After stimulation, media was removed, and LSECs were co-cultured in indirect contact via inserts with MEECs or AM for 48 hours. Then, LSECs were resuspended in TRIzol reagent for RNA isolation and analysis of adhesion molecules gene expression by Real-Time PCR. Experiments were performed in triplicates in two independent experiments and reported as mean ± SD.

RNA isolation and gene expression analysis by Real-Time PCR

Cells from *in vitro* experiments were lysed in TRIzol™ reagent, and total RNA was extracted according to the manufacturer's protocol. Briefly, chloroform (Sigma-Aldrich) was added, and the samples were incubated for 5 minutes and then centrifuged (15 minutes at 12,000 × g at 4°C). The mixture separated into a lower phenol-chloroform, an interphase, and a colorless upper aqueous phase. The aqueous phase containing the RNA was transferred to a new tube, and isopropanol (Sigma-Aldrich) and glycogen (#R0551, Thermo Scientific) was added to precipitate the RNA. The RNA was then washed with 75% ethanol (PanReac AppliChem) and resuspended in RNase-free water. Total RNA from the liver was extracted using commercially available kits: RNeasy

(Gibco-Invitrogen, Paisley, UK). A 1 µg or 0.5 µg aliquot of total RNA was reverse transcribed using a complementary DNA synthesis kit (High-Capacity cDNA Reverse Transcription Kit, Applied Biosystems, Foster City, California, USA). Primers and probes for human and mice gene expression assays (Applied Biosystems) were selected as follows: CYP2B6 (Taqman assay reference from Applied Biosystems: Human: Hs04183483_g1; Mouse: Mm00657910_m1), HGF (Human: Hs00300159_m1; Mouse: Mm01135184_m1), NOS2 (Human: Hs01075529_m1; Mouse: Mm00440502_m1), TNF-α (Human: Hs00174128_m1; Mouse: Mm00443258_m1), ARG1 (Human: Hs00163660_m1; Mouse: Mm00475988_m1), MRC1 (Human: Hs00267207_m1; Mouse: Mm00485148_m1), ICAM-1 (Human: Hs00164932_m1; Mouse: Mm00516023_m1), VCAM-1 (Human: Hs01003372_m1; Mouse: Mm01320970_m1), CX3CL1 (Hs00171086_m1), CXCL16 (Hs00222859_m1), MMP-2 (Human: Hs01548727_m1; Mouse: Mm00439498_m1), MMP-9 (Human: Hs00957562_m1; Mouse: Mm00442991_m1), COX-2 (Mm00478374_m1), IL-1β (Mm00434228_m1), RETNLA (Mm00445109_m1), E-selectin (Hs00174057_m1), TGF-β (Mm01178820_m1), PDGF-BB (Mm00440677_m1), OSM (Mm01193966_m1), COL1A1 (Mm00801666_g1), TIMP-1 (Mm01341360_g1), α-SMA (Mm01204962_gH) and human glyceraldehyde-3-phosphate dehydrogenase (GAPDH: Hs02786624_g1) and hypoxanthine phosphoribosyl transferase (HPRT: Mm03024075_m1) used as endogenous standard. Expression assays were designed using the Taqman Gene Expression assay software (Applied Biosystems). Real-time quantitative PCR was performed in duplicates with a Lightcycler-480 II (Roche Diagnostics). A 10 µl of diluted 1:8 cDNA, Taqman probe and primers and FastStart TaqMan Master (Applied Biosystems) was used in each PCR. The fluorescence signal was captured during each of the 45 cycles (denaturing 10s at 95°C, annealing 15s at 60°C

and extension 20s at 72°C). Water was used as a negative control. Relative quantification was calculated using the comparative threshold cycle (CT), which is inversely related to the abundance of mRNA transcripts in the initial sample. The mean CT of duplicate measurements was used to calculate ΔCT as the difference in CT for target and reference. The relative quantity of the product was expressed as fold induction of the target gene compared with the control primers according to the formula $2^{-\Delta\Delta CT}$, where ΔΔCT represents ΔCT values normalized with the mean ΔCT of control samples.

Immunofluorescent staining in liver tissue

Liver was excised, washed with PBS, and fixed with 10% buffered formaldehyde solution for 24h. Afterwards, the tissue was embedded in paraffin. Before staining, paraffin was removed using xylene, ethanol, and deionized water. Liver sections (4 µm) underwent antigen retrieval in 1% SDS solution for 20 minutes at room temperature and were then blocked with 5% normal horse serum. Liver sections from fibrotic and fibrotic hepatectomized mice were incubated with goat polyclonal anti-PCNA (1:50, Sigma, ref: SAB2502098-100UG) overnight at 4ºC and revealed with Cy3-conjugated donkey-anti-goat IgG (Jackson ImmunoResearch Laboratories, ref: 705-165-147) incubated for 1h at room temperature. Liver sections obtained from the interface between hepatectomized liver and the implant of MEECs were stained with the specific marker CD32b (1:200, Santa Cruz Biotechnology, ref: sc-365864) and human endothelial cells from MEECs with rhodamine Ulex europaeus agglutinin-1 (UEA-1, Vector Laboratories, ref: RL-1062-2). The presence of PCNA was visualized in an epifluorescence microscope. DAPI (Fluoroshield, Sigma) was used to counterstain cell nuclei. Negative controls of immunofluorescence staining were prepared by incubating liver sections with the corresponding Cy3 secondary antibody without the incubation of the primary antibody.

Sirius Red staining and Fibrosis quantification

Liver was excised, washed with PBS, and fixed with 10% buffered formaldehyde solution for 24h. Afterwards, the tissue was embedded in paraffin. Before staining, paraffin was removed using xylene, ethanol, and deionized water. Liver sections (4 µm) were stained in 0.1% Sirius Red F3B (Sigma) with saturated picric acid (Sigma). Relative fibrosis area (expressed as a percentage of total liver area) was analyzed in 20 fields of Sirius red-stained liver sections per animal using the morphometry software ImageJ version 1.53c. To evaluate the relative fibrosis area, the measured collagen area was divided by the net field area and then multiplied by 100. The extent of fibrosis was measured as percentage for each animal, and the average value was presented.

Hydroxyproline measurement in liver

Liver hydroxyproline content was measured following the manufacturer's protocol (Hydroxyproline Assay Kit, Sigma-Aldrich). Briefly, 10 mg of liver tissue was homogenized in distilled water, mixed with an equal volume of 10 M concentrated sodium hydroxide, and incubated at 120°C for 1 h. After alkaline hydrolysis, the samples were neutralized, oxidized, and then treated with 4-dimethylaminobenzaldehyde (DMAB) solution. Sample absorbance was measured at 560 nm in duplicate. Hydroxyproline content was expressed at microgram of hydroxyproline per gram liver.

Western Blot

Total protein was extracted from cells with Lysis buffer solution containing 20 mM Tris-HCl, pH 7.4, 1% Triton X-100, 0.1% SDS, 50 mM NaCl, 2.5 mM EDTA, 1 mM $Na_4P_2O_7$ $10H_2O$, 20 mM NaF, 1 mM Na_3VO_4 2 mM, 2 mM Pefabloc and a cocktail of protease inhibitors (Complete Mini, Roche). Proteins were separated on a 10% SDS polyacrylamide gel (Mini Protean III, BioRad) and transferred for 7 min to nitrocellulose membranes using the Trans-Blot® Turbo™ Transfer System and Transfer Stacks

(Biorad). As a loading control, nitrocellulose membranes were stained with Ponceau-S red. Membranes were then blocked for 1 hour with 5 % powdered defatted milk in TPBS buffer and were incubated overnight at 4°C with rabbit anti-HGF polyclonal antibody (1:1000, Abcam, ref: ab24865), rabbit anti-FGF2 polyclonal antibody (1:1000, Thermo Fisher, ref: PA5-116495) or mouse anti-HS antibody (1:1000, Sigma-Aldrich, ref: MAB2040) and β-actin (1:2000, Cell Signaling, ref: 4970) as loading control. Following primary antibody incubation, membranes were incubated with a horseradish peroxidase (HRP) conjugated donkey anti-rabbit secondary antibody (1:2000, Thermo Fisher, ref: SA1-200) or HRP conjugated donkey anti-mouse secondary antibody (1:2000, Jackson ImmunoResearch Laboratories, ref: 715-035-150) for 1 hour at room temperature. Bands were visualized by chemiluminescence with SuperSignal™ West Pico PLUS Chemiluminescent Substrate (Thermo Scientific, Waltham, MA, USA) using a ChemiDoc Imaging System (Biorad Laboratories, Inc) and quantified by computer-assisted densitometry analysis (ImageJ).

Supplementary figures

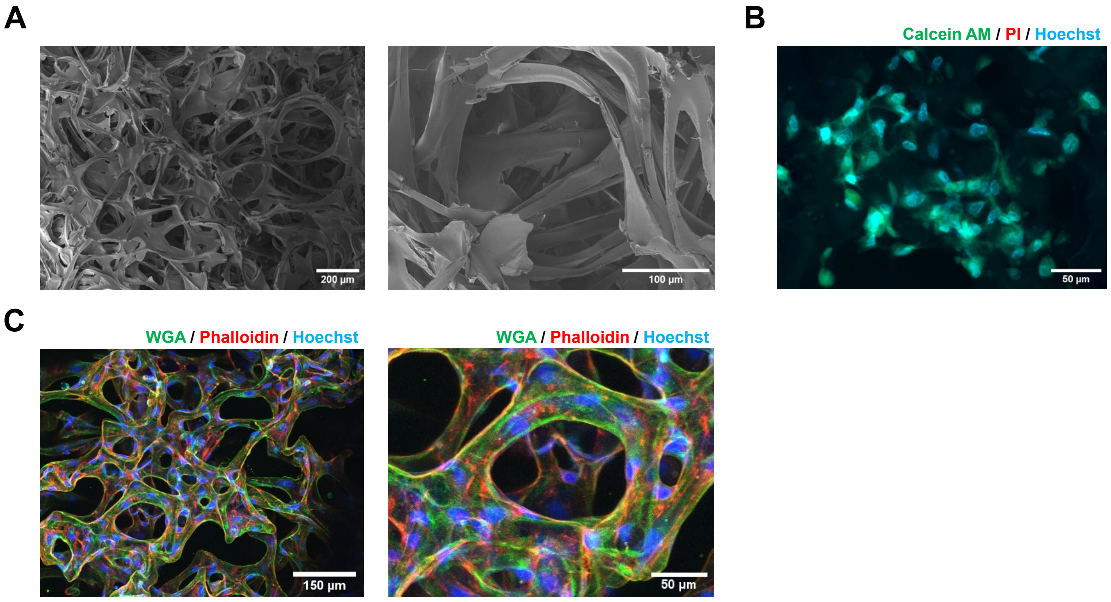

Fig. S1. Characterization of compressed denatured 3D collagen matrices and matrix-embedded endothelial cells (MEECs). (A) Scanning Electron Microscopy (SEM) images of the porous structure of compressed denatured 3D collagen matrices (Gelfoam®) at different magnifications. **(B)** Z-projection confocal images of MEECs stained with Calcein AM (green), propidium iodide (PI, red) and Hoechst (blue). **(C)** Z-projection confocal images of MEECs stained with Wheat Gern Agglutinin (WGA, green), Phalloidin (red) and Hoechst (blue) at different magnifications.

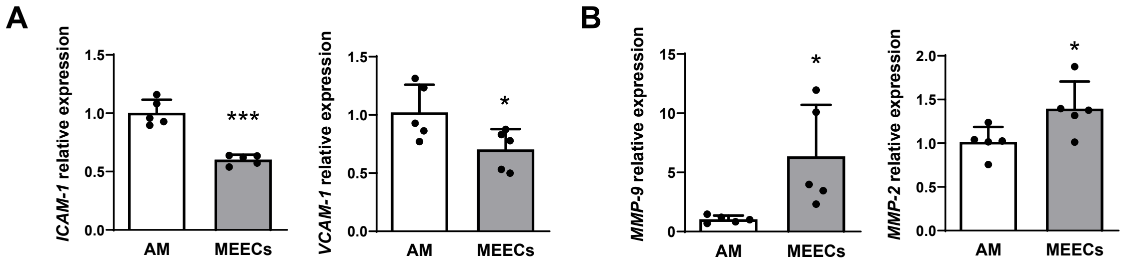

Fig. S2. Perihepatic implantation of MEECs downregulates the expression of adhesion molecules and upregulates the expression of metalloproteinases. (A) Hepatic expression of the intracellular adhesion molecule (ICAM, $p < 0.0001$, student's t test) and vascular cell adhesion molecule (VCAM, $p = 0.0425$, student's t test) in fibrotic mice receiving AM or MEECs implants. (B) Hepatic expression of metalloproteinase 9 (MMP-9, $p = 0.0259$, student's t test) and metalloproteinase 2 (MMP-2, $p = 0.0429$, student's t test) in fibrotic mice receiving AM or MEECs implants. $n = 5$ animals per group. Data are shown as means ± SD. *$p \leq 0.05$, and **$p \leq 0.01$, student's t test.

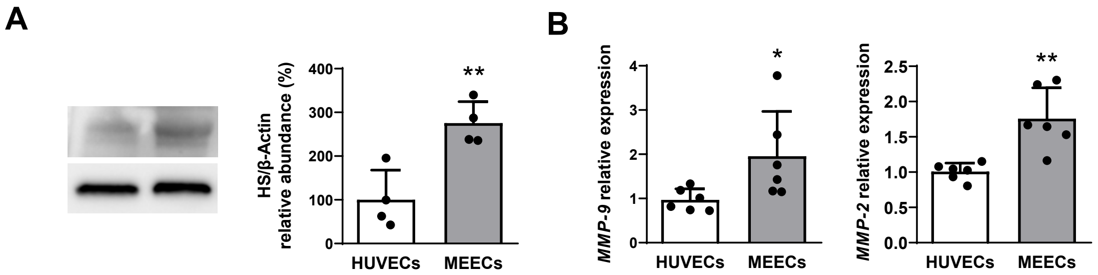

Fig. S3. Matrix embedding of endothelial cells maximizes protein and gene expression of anti-fibrotic factors and ECM modulators. (A) Western Blot analysis of heparan sulfate (HS) and β-actin in endothelial cells (ECs) in 2D cultures (2D-ECs) or embedded in collagen 3D matrices (MEECs) and HS to β-actin relative protein abundance (%) ($p = 0.0058$, student's t test). $n = 4$. **(B)** Gene expression of the metalloproteinase 9 ($p = 0.0436$, student's t test) and metalloproteinase 2 ($p = 0.0023$, student's t test) in 2D-ECs and MEECs. $n = 6$. Data are shown as means ± SD. *$p \leq 0.05$, and ** $p \leq 0.01$, student's t test.

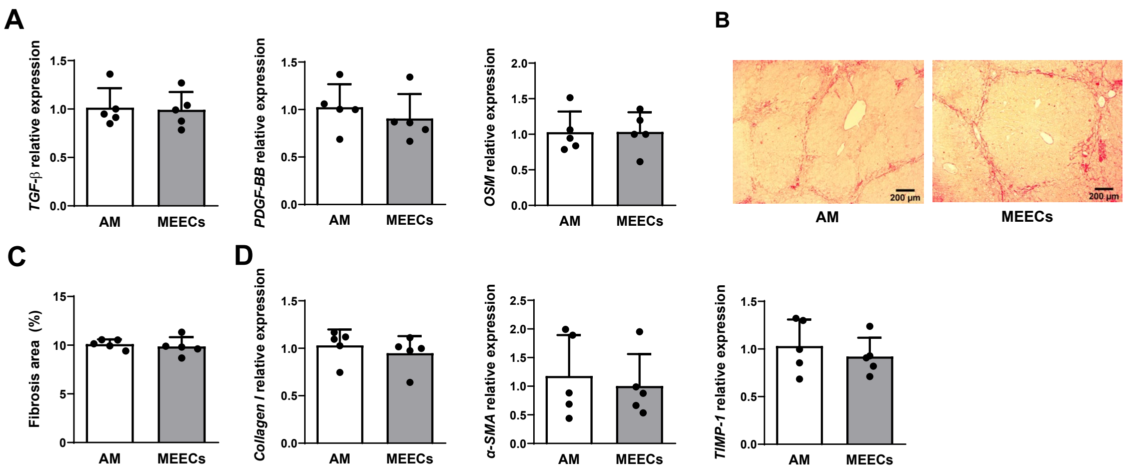

Fig. S4. Subcutaneous implantation of MEECs does not reduce fibrosis in fibrotic mice. (A) Hepatic expression of the macrophage-derived HSC activators transforming growth factor-beta (TGF-β, p = 0.8565, student's t test), platelet-derived growth factor-BB (PDGF-BB, p = 0.4760, student's t test), and oncostatin M (OSM, p = 0.9844, student's t test) in fibrotic mice treated with subcutaneous AM or MEECs implants for one week. **(B)** Sirius red staining (p = 0.6218, student's t test). **(C)** Quantification of liver fibrosis area in fibrotic mice treated with subcutaneous AM or MEECs implants. **(D)** Hepatic expression of the extracellular matrix turnover genes Collagen-I (Col-I, p = 0.4718, student's t test), alpha-smooth muscle actin (α-SMA, p = 0.6772, student's t test) and tissue inhibitor of metalloproteinases-1 (TIMP-1, p = 0.4892, student's t test) in fibrotic mice treated with subcutaneous AM or MEECs implants. n = 5 animals per group. Data are shown as means ± SD.

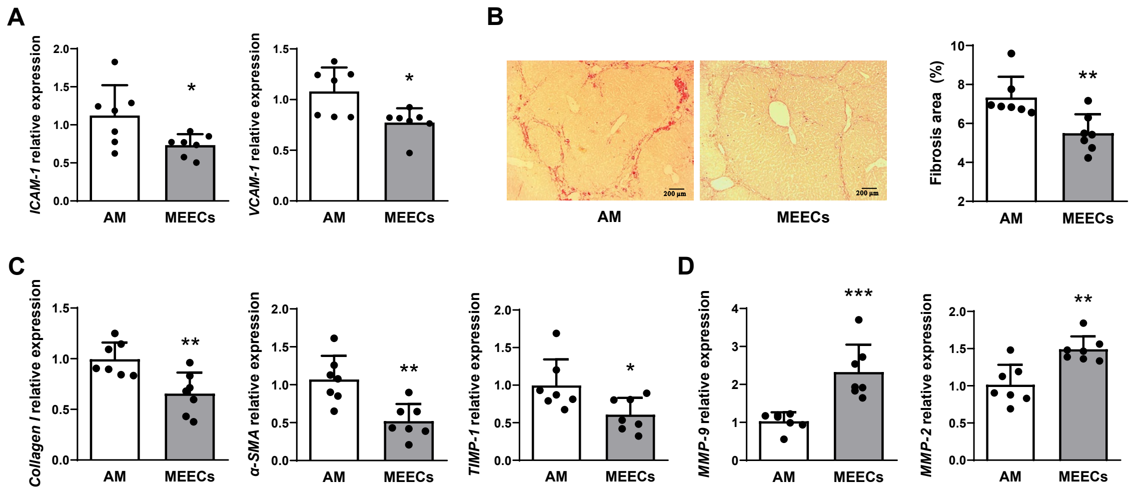

Fig. S5. Perihepatic implantation of MEECs downregulates the hepatic expression of adhesion molecules and reduces fibrosis in a fibrotic hepatectomized mice. (A) Hepatic expression of the intracellular adhesion molecule 1 (ICAM-1, student's *t* test, $p = 0.0320$) and the vascular adhesion molecule (VCAM-1, $p = 0.0122$, student's *t* test) in fibrotic hepatectomized mice treated with perihepatic AM or MEECs implants. **(B)** Sirius red staining and quantification of liver fibrosis area ($p = 0.0058$, student's *t* test). **(C)** Hepatic expression of the extracellular matrix turnover genes Collagen-I (Col-I, $p = 0.0055$, student's *t* test), alpha-smooth muscle actin (α-SMA, $p = 0.0027$, student's *t* test) and tissue inhibitor of metalloproteinases-1 (TIMP-1, $p = 0.0285$, student's *t* test) in fibrotic hepatectomized mice treated with perihepatic AM or MEECs implants. **(D)** Hepatic expression of metalloproteinase 9 (MMP-9, $p = 0.0007$, student's *t* test) and 2 (MMP-2, $p = 0.0019$, student's *t* test) in fibrotic hepatectomized mice treated with perihepatic AM or MEECs implants. $n = 7$ animals per group. Data are shown as means ± SD. $^{*}p \leq 0.05$, and $^{**}p \leq 0.01$, student's t test.

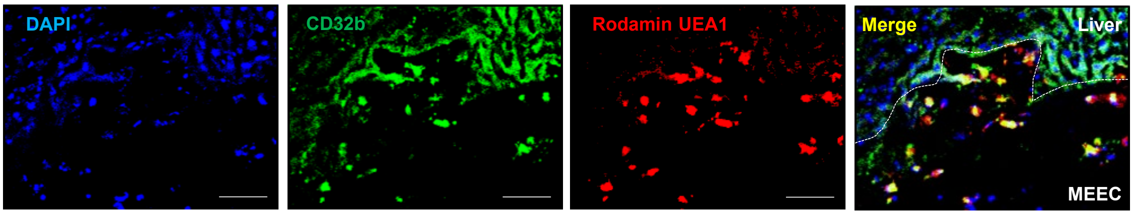

Fig. S6. Endothelial cells embedded in matrices in close contact with hepatectomized liver undergo a phenotype switch to LSEC. Immunofluorescence staining of LSECs with their specific marker CD32b and human endothelial cells in MEECs with rhodamine Ulex europaeus agglutinin-1 (UEA-1) in the livers of fibrotic hepatectomized mice receiving a MEECs perihepatic implant. Scale bars 50 µm.

Supplementary table

Table S1: Demographic and baseline characteristics of study participants.

**Variables**	**Cirrhotic**<br>n=8
**Age (years)**	61.3 ± 7
**Gender**	
**Male**	7 (27.5)
**Female**	1 (6.5)
**BMI (kg/m^2)**	27.5 ± 6.5
**Etiology of liver disease**	
**Alcoholic**	5 (62.5)
**Alcoholic + MASLD**	1 (12.5)
**Alcoholic + ACLF II**	1 (12.5)
**MASLD**	1 (12.5)
**Child-Pugh Score**	
**B**	3 (37.5)
**C**	5 (62.5)
**MELD score**	21.5 ± 2.6
**Cirrhosis duration (years)**	4.2 ± 4.0

Data is shown as Mean ± S.D., or Number of Participants (Percentage, %).

BMI: Body Mass Index; MASLD: Metabolic associated steatotic liver disease; ACLF II: Acute-on-Chronic Liver Failure; MELD: Model for End-Stage Liver Disease
