# Supplementary material for: 3D endothelial cell scaffolds protect liver explants and exhibit therapeutic effects on liver fibrosis
Source: JHEP Rep. 2025 Oct 4;8(1):101617. doi: 10.1016/j.jhepr.2025.101617 (PMC12890448; doi:10.1016/j.jhepr.2025.101617)
Supplement: Multimedia component 2 [file mmc2.pdf]

JHEP|Reports

JHEP Reports

CTAT methods

Tables for a "Complete, Transparent, Accurate and Timely account" (CTAT) are now mandatory for all revised submissions. The aim is to enhance the reproducibility of methods.

- Only include the parts relevant to your study
- Refer to the CTAT in the main text as 'Supplementary CTAT Table'
- Do not add subheadings
- Add as many rows as needed to include all information
- Only include one item per row

If the CTAT form is not relevant to your study, please outline the reasons why:

1.1 Antibodies

Name	Citation	Supplier	Cat no.	Clone no.
Rabbit anti-HGF polyclonal antibody		Abcam	ab24865	Polyclonal
Rabbit anti-FGF2 polyclonal antibody		Thermo Fisher	PA5-116495	Polyclonal
Mouse Anti-Heparin/Heparan Sulfate Antibody		Sigma-Aldrich	MAB2040	T320.11
β-Actin (13E5) Rabbit mAb		Cell Signaling	4970	13E5
Donkey anti-Rabbit IgG (H+L) Cross-Adsorbed Secondary Antibody, HRP		Thermo Fisher	SA1-200	
Peroxidase AffiniPure® Donkey Anti-Mouse IgG (H+L)		Jackson ImmunoResearch Laboratories	715-035-150	
Anti-PCNA antibody produced in goat		Sigma-Aldrich	SAB2502098	Polyclonal
Cy™3 AffiniPure™ Donkey Anti-Goat IgG (H+L)		Jackson ImmunoResearch Laboratories	705-165-147	Polyclonal
CD32-B antibody		Santa Cruz Biotechnology	sc-365864	F-4

1.2 Cell lines

Name	Citation	Supplier	Cat no.	Passage no.	Authentication test method
HUV-EC-C [HUVEC]	Melgar-Lesmes P, Balcells M, Edelman ER. Implantation of healthy matrix-embedded endothelial cells rescues dysfunctional endothelium and ischaemic tissue in liver engraftment. Gut. 66(7):1297-1305 (2017). doi: 10.1136/gutjnl-2015-310409.	ATCC	CRL-1730		
Human Hepatic Sinusoidal Endothelial Cells	Dirscherl, K., Schläpfer, M., Roth Z'graggen, B. et al. Hypoxia sensing by hepatic stellate cells leads to VEGF-dependent angiogenesis and may contribute to accelerated liver regeneration. Sci Rep 10, 4392 (2020). doi: 10.1038/s41598-020-60709-9	Innoprot	P10652		

1.3 Organisms

Name	Citation	Supplier	Strain	Sex	Age	Overall n number
Mus Musculus		Charles River Laboratories	Balb/c	male	7-week	57

1.4 Sequence based reagents

Name	Sequence	Supplier

1.5 Biological samples

Description	Source	Identifier
Human cirrhotic liver samples obtained from patients with end-stage cirrhosis	Liver Transplant Unit, Institut Clínic de Malalties Digestives i Metabòliques (ICMDM), Hospital Clínic, Barcelona, Spain.	

1.6 Deposited data

Name of repository	Identifier	Link

1.7 Software

Software name	Manufacturer	Version
GraphPad Prism	GraphPad Software, Boston, MA, USA.	8.0.1
Image J		1.54p
Fiji		2.16.0

1.8 Other (e.g. drugs, proteins, vectors etc.)

Ultrapure™ Low Melting Point Agarose	Invitrogen	16520100
Calcein AM	Sigma-Aldrich	206700
Propidium Iodide	Sigma-Aldrich	537059
Hoechst 33342	Invitrogen	H3570
Wheat Germ Agglutinin	Invitrogen	W11261
Alexa Fluor Plus 555 Phalloidin	Invitrogen	A30106
ATP Bioluminescence Assay Kit CLS II	Roche	11699695001
Lipopolysaccharides (LPS) from Escherichia coli O127:B8	Sigma-Aldrich	L3129
Histodenz™ nonionic density gradient medium	Sigma-Aldrich	D2158
CD11b MicroBeads, human and mouse	Miltenyi Biotec	130-049-601
TRIzol reagent	Invitrogen	15596026
NucleoSpin® RNA kit	Macherey Nagel	740955
High-Capacity cDNA Reverse Transcription Kit	Applied Biosystems	4368814
TaqMan Gene Expression Assay CYP2B6 (Human)	Applied Biosystems	Hs04183483_g1
TaqMan Gene Expression Assay CYP2B6 (Mouse)	Applied Biosystems	Mm00657910_m1
TaqMan Gene Expression Assay HGF (Human)	Applied Biosystems	Hs00300159_m1
TaqMan Gene Expression Assay HGF (Mouse)	Applied Biosystems	Mm01135184_m1
TaqMan Gene Expression Assay NOS2 (Human)	Applied Biosystems	Hs01075529_m1
TaqMan Gene Expression Assay NOS2 (Mouse)	Applied Biosystems	Mm00440502_m1
TaqMan Gene Expression Assay TNF-α (Human)	Applied Biosystems	Hs00174128_m1
TaqMan Gene Expression Assay TNF-α (Mouse)	Applied Biosystems	Mm00443258_m1
TaqMan Gene Expression Assay ARG1 (Human)	Applied Biosystems	Hs00163660_m1
TaqMan Gene Expression Assay ARG1 (Mouse)	Applied Biosystems	Mm00475988_m1
TaqMan Gene Expression Assay MRC1 (Human)	Applied Biosystems	Hs00267207_m1
TaqMan Gene Expression Assay MRC1 (Mouse)	Applied Biosystems	Mm00485148_m1
TaqMan Gene Expression Assay ICAM-1 (Human)	Applied Biosystems	Hs00164932_m1
TaqMan Gene Expression Assay ICAM-1 (Mouse)	Applied Biosystems	Mm00516023_m1
TaqMan Gene Expression Assay VCAM-1 (Human)	Applied Biosystems	Hs01003372_m1
TaqMan Gene Expression Assay VCAM-1 (Mouse)	Applied Biosystems	Mm01320970_m1
TaqMan Gene Expression Assay CX3CL1 (Human)	Applied Biosystems	Hs00171086_m1
TaqMan Gene Expression Assay CXCL16 (Human)	Applied Biosystems	Hs00222859_m1
TaqMan Gene Expression Assay MMP-2 (Human)	Applied Biosystems	Hs01548727_m1
TaqMan Gene Expression Assay MMP-2 (Mouse)	Applied Biosystems	Mm00439498_m1
TaqMan Gene Expression Assay MMP-9 (Human)	Applied Biosystems	Hs00957562_m1

TaqMan Gene Expression Assay MMP-9 (Mouse)	Applied Biosystems	Mm00442991_m1
TaqMan Gene Expression Assay COX-2 (Mouse)	Applied Biosystems	Mm00478374_m1
TaqMan Gene Expression Assay IL-1β (Mouse)	Applied Biosystems	Mm00434228_m1
TaqMan Gene Expression Assay RETNLA (Mouse)	Applied Biosystems	Mm00445109_m1
TaqMan Gene Expression Assay E-selectin (Human)	Applied Biosystems	Hs00174057_m1
TaqMan Gene Expression Assay TGF-β (Mouse)	Applied Biosystems	Mm01178820_m1
TaqMan Gene Expression Assay PDGF-BB (Mouse)	Applied Biosystems	Mm00440677_m1
TaqMan Gene Expression Assay OSM (Mouse)	Applied Biosystems	Mm01193966_m1
TaqMan Gene Expression Assay COL1A1 (Mouse)	Applied Biosystems	Mm00801666_g1
TaqMan Gene Expression Assay TIMP-1 (Mouse)	Applied Biosystems	Mm01341360_g1
TaqMan Gene Expression Assay α-SMA (Mouse)	Applied Biosystems	Mm01204962_gH
TaqMan Gene Expression Assay GAPDH (Human)	Applied Biosystems	Hs02786624_g1
TaqMan Gene Expression Assay HPRT (Mouse)	Applied Biosystems	Mm03024075_m1
Rhodamine Ulex europaeus agglutinin 1	Vector Laboratories	RL-1062-2
Fluoroshield™ with DAPI	Sigma-Aldrich	F6057
Direct Red 80 Dye content 25 %	Sigma-Aldrich	365548
Picric acid solution 1.3% in H2O	Sigma-Aldrich	P6744
Hydroxyproline Assay Kit	Sigma-Aldrich	MAK357-1KT

1.9 Please provide the details of the corresponding methods author for the manuscript:

Corresponding author: Pedro Melgar-Lesmes, PhD.

Department of Biomedicine, School of Medicine, University of Barcelona.

C/ Casanova 143, 08036, Barcelona, Spain.

Phone: +34 934020294
E-mail: pmelgar@ub.edu.

2.0 Please confirm for randomised controlled trials all versions of the clinical protocol are included in the submission. These will be published online as supplementary information.

This study did not involve clinical randomised controlled trials.
